# Biodiversity patterns of the coral reef cryptobiota around the Arabian Peninsula

**DOI:** 10.1038/s41598-024-60336-8

**Published:** 2024-04-25

**Authors:** Rodrigo Villalobos, Eva Aylagas, John K. Pearman, Joao Curdia, Darren Coker, Alyssa Clothilde Bell, Shannon D. Brown, Katherine Rowe, Diego Lozano-Cortés, Lotfi J. Rabaoui, Alyssa Marshell, Mohammad Qurban, Burton Jones, Michael Lee Berumen, Susana Carvalho

**Affiliations:** 1 King Abdullah University of Science and Technology (KAUST), Red Sea Research Center, 23955-6900 Thuwal, Kingdom of Saudi Arabia; 2 Coastal and Freshwater Group, Cawthron Institute, Nelson, New Zealand; 3 Department of Biology, University of Konstanz, Konstanz, Germany; 4 Cooperative Institute for Climate, Ocean, and Ecosystem Studies, University of Washington, Seattle, WA USA; 5 School of Science, The University of Waikato, Hamilton, New Zealand; 6 Environmental Protection, Saudi Aramco, Dhahran, Kingdom of Saudi Arabia; 7 Center for Environment & Marine Studies, Research Institute, King Fahd University of Petroleum and Minerals, 31261 Dhahran, Eastern Province Kingdom of Saudi Arabia; 8 National Center for Wildlife, Riyadh, Saudi Arabia; 9 Sultan Qaboos University, Al Seeb Al Khoudh SQU SEPS, 123 Muscat, Oman; 10 Institute for Marine and Antarctic Studies, University of Tasmania, Hobart, TAS 7053 Australia; 11 Ministry of Environment, Water and Agriculture, Riyadh, Saudi Arabia

**Keywords:** Biogeography, ARMS, Red Sea, Arabian (Persian) Gulf, Gulf of Oman, Coelobites, Cryptofauna, Distribution patterns, Biodiversity, Community ecology, Ecology, Coral reefs

## Abstract

The Arabian Peninsula accounts for approximately 6% of the world’s coral reefs. Some thrive in extreme environments of temperature and salinity. Using 51 Autonomous Reef Monitoring Structure (ARMS), a standardized non-destructive monitoring device, we investigated the spatial patterns of coral reef cryptobenthic diversity in four ecoregions around the Arabian Peninsula and analyzed how geographical and/or environmental drivers shape those patterns. The mitochondrial cytochrome c oxidase subunit I (COI) gene was used to identify Amplicon Sequence Variants and assign taxonomy of the cryptobenthic organisms collected from the sessile and mobile fractions of each ARMS. Cryptobenthic communities sampled from the two ecoregions in the Red Sea showed to be more diverse than those inhabiting the Arabian (Persian) Gulf and the Gulf of Oman. Geographic distance revealed a stronger relationship with beta diversity in the Mantel partial correlation than environmental distance. However, the two mobile fractions (106–500 µm and 500–2000 µm) also had a significant correlation between environmental distance and beta diversity. In our study, dispersal limitations explained the beta diversity patterns in the selected reefs, supporting the neutral theory of ecology. Still, increasing differences in environmental variables (environmental filtering) also had an effect on the distribution patterns of assemblages inhabiting reefs within short geographic distances. The influence of geographical distance in the cryptofauna assemblages makes these relevant, yet usually ignored, communities in reef functioning vulnerable to large scale coastal development and should be considered in ecosystem management of such projects.

## Introduction

The coral reefs of the Arabian Peninsula account for approximately 6% of the world’s coral reef cover^1^ and exhibit high levels of endemism^2,3^. The Arabian (Persian) Gulf and the Red Sea have been used as model ecosystems to understand the biological components of reefs under extreme environments and along natural environmental gradients, respectively^4,5^. However, the Arabian Peninsula coral reefs are relatively understudied in comparison to those on the Great Barrier Reef and in the Caribbean^6,7^, although this gap in knowledge has been closing recently^7^. Coral reefs in the Arabian Peninsula experience a variety of conditions dictated by various environmental parameters and differences in geological and paleo-climatic histories. The Red Sea contains continuous fringing reefs along most of its coastline with cross-shelf reef complexes at present in the center of the Red Sea, while in the southern Red Sea, there are patch and barrier reefs as well as island complexes^8^. The macrobenthic community of coral reefs is homogenous in the northern and central Red Sea offshore reefs and is dominated by hard corals, coralline algae, turf algae, and soft corals^9,10^. However, in the central Red Sea, differences occur in the benthic communities with distance from shore, with decreased coral cover in inshore reefs and changing relative abundance of various genera^11^. The coral reefs in the southern Red Sea present a distinct benthic community dominated by macroalgae^10^, possibly given the high turbidity and productivity in the area^8,9^.

Currently, the Red Sea contains 359 coral species^8^, where *Pocillopora*, *Millepora*, and *Porites* are generally the dominant genera^9^. The Omani side of the Gulf of Oman has coral reefs in three locations: the Musadan Peninsula, the Daymaniyat Islands, and the Muscat area^12^. The coral reefs in the Gulf of Oman harbor 117 species of zooxanthellate corals^8^, with more diverse coral assemblages in the north at the Musadan Peninsula^12^. Coral assemblages in the north of Oman are dominated by Poritidae, Favidae, and Pocilloporidae^13^. The Arabian Gulf coral reefs are distributed primarily in reefs referred to as ‘coral carpets’, where colonies grow on exposed rock and are primarily present in the United Arab Emirates and islands in the center of the Arabian (Persian) Gulf^5,14^. The Arabian (Persian) Gulf reefs have 66 species of corals, dominated by families Poritidae and Merulinidae^8,15^. Every region experienced bleaching events that did not occur simultaneously; however, the absence of established long-term monitoring programs may have led to some of these events going unnoticed. The Red Sea has documented bleaching events in the summers of 2010 and 2015^16,17^, whereas the Gulf of Oman suffered a bleaching event in 2018^18^. The Arabian (Persian) Gulf suffered from a bleaching event in 2017^19^. These bleaching events have been known to change the composition of reefs in the region^17^, but the main differences between regions should still persist.

Differences in biodiversity around the Arabian Peninsula has been shown for a range of fauna, including annelids, arthropods, corals, fishes, other chordates, echinoderms, and mollusks^2^. Inter-basin comparisons around the Arabian Peninsula have provided a broader understanding of the coral reefs in the region. Coker et al.^20^ found the Red Sea to have a more diverse and abundant cryptobenthic fish community than what had been reported for the Gulf of Oman and the Arabian (Persian) Gulf^21^. Investigations have also shown differences between the northern and central Red Sea and the southern Red Sea for plankton^22,23^ as well as in the reef cryptobenthic communities^10,24^. However, further efforts are needed to fully understand the biodiversity patterns around the Arabian Peninsula, especially for reef communities not targeted in visual reef surveys.

**Figure 1:**
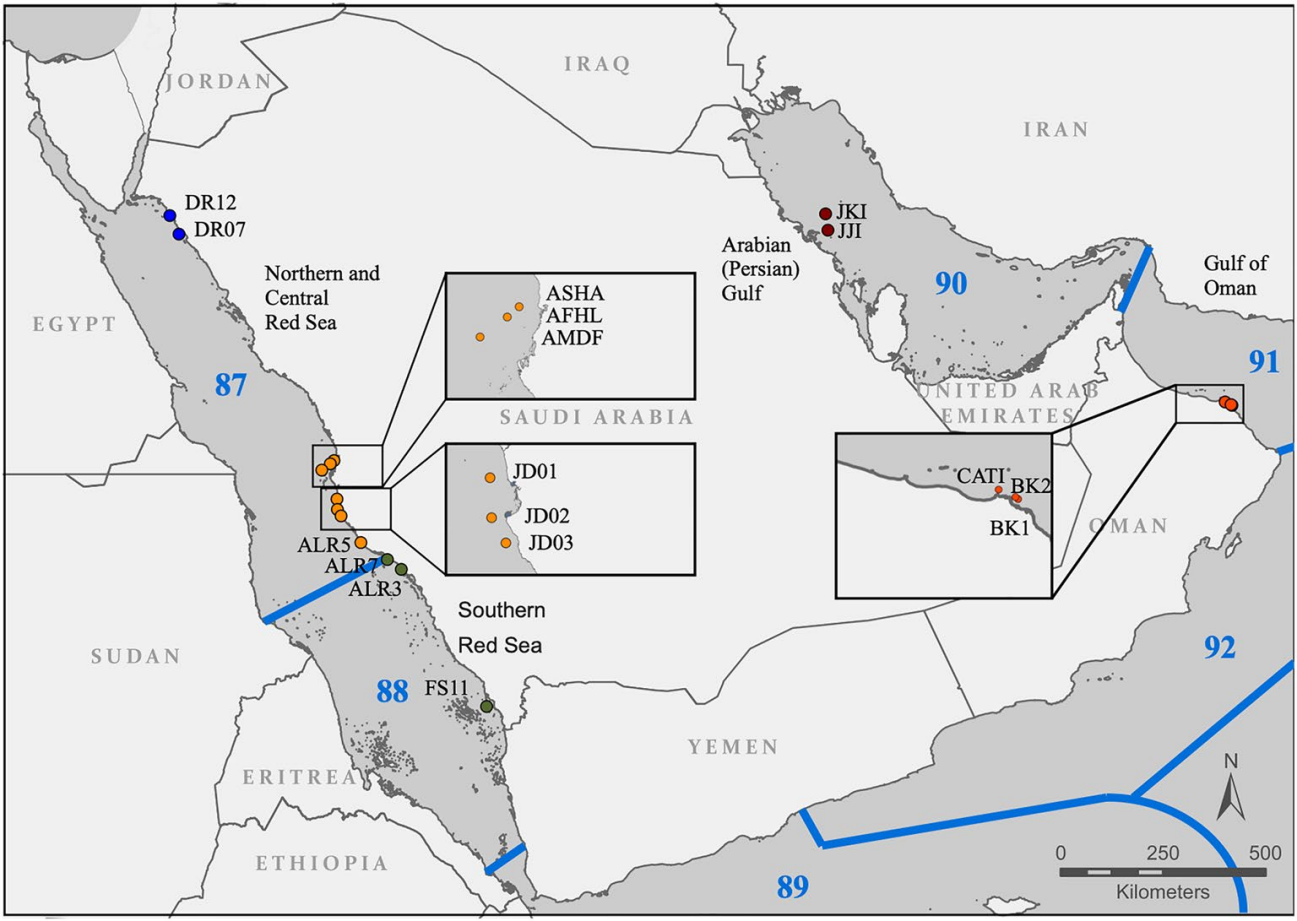
Locations of the reefs where ARMS were deployed around the Arabian Peninsula by region. In blue the northern Red Sea region, in orange the central Red Sea region, in green the southern Red Sea region, in maroon the Arabian (Persian) Gulf, and in red the Gulf of Oman. Numbers in blue refer to the marine ecoregions according to Spalding et al., 2007^25^ delineated in the same color. 87 is the Northern and Central Red Sea ecoregion, 88 is the Southern Red Sea ecoregion, 89 is the Gulf of Aden ecoregion, 92 is the Western Arabian Sea ecoregion, 91 is the Gulf of Oman ecoregion, and 90 is the Arabian (Persian) Gulf ecoregion. Numbers are congruent with the original numbering of the ecoregions^25^.

The Arabian Peninsula is divided into two provinces and six ecological regions due to variations in species composition and paleoclimatic histories^25^. One of the provinces includes the Red Sea and Gulf of Aden, which is further subdivided into the Northern and Central Red Sea, Southern Red Sea, and Gulf of Aden ecological regions. The second province is the Somali/Arabian province, comprising the Arabian (Persian) Gulf, Gulf of Oman, and Western Arabian Sea ecological regions^25^ (Fig. 1). However, a recent proposal suggests a further refinement of these regions^9^. Four of the ecological regions proposed by Spalding et al.^25^ were represented in this study (two in the Red Sea, the Arabian (Persian) Gulf, and the Gulf of Oman), with vastly different environmental conditions. The two Red Sea regions are characterized by a latitudinal gradient for both temperature and salinity, with the north region being colder and more saline than the south region^26^, which is generally more productive^27^. The Red Sea also experiences seasonal upwelling events that bring nutrients into the surface waters^28,29^. The Red Sea has experienced intermittent periods of partial isolation from the Indian Ocean and increased salinity during glacial maxima, and currently, the connection with the Indian Ocean is still restricted through the strait Bab al Mandab^30^. Along with the natural environmental barrier provided by the upwelling outside the Gulf of Aden and the comparatively higher primary productivity^31^, these conditions may have contributed to the large numbers of endemic species in the area^31^. The third ecological region (i.e., the Arabian (Persian) Gulf) is a shallow water body (< 100 m) with restricted water flow with the Indian Ocean through the Strait of Hormuz. These characteristics result in more extreme oscillations in the sea surface temperature that can reach a variation of 20 °C between seasons. The fourth region represented here is the Gulf of Oman, where the environmental conditions are a result of the southwest and northeast monsoon seasons^32^. In general, the Gulf of Oman has a higher productivity and lower temperatures below the surface due to strong upwelling^32,33^ compared to the other regions, although the surface waters can be 10 °C warmer than below the thermocline^34^. These conditions are unique around the Arabian Peninsula, particularly considering the four regions analyzed, which may act as a natural barrier to species colonization^35^.

Most of the mobile and sessile biodiversity on reefs often go unseen as they are composed of organisms that can be small, well camouflaged, nocturnal, or hidden in crevices within the reef matrix^36^. These are formally named the cryptobiome and comprise a wide variety of groups like crustaceans, annelids, and fish^24^ but also bacteria^37^. Despite a growing body of literature focused on this fundamental biodiversity component in the region^20,24,37–44^, a comparative assessment of biodiversity patterns across the multiple ecoregions around the Arabian Peninsula is missing. The development of tools and standardized methods in the last decade have promoted concerted and unprecedented efforts at the global scale to disentangle patterns of change in the cryptobiome^38,45–47^. The Autonomous Reef Monitoring Structure (ARMS) developed during the Census of Marine Life provides a standardized and non-destructive method to mimic the structural complexity of the reef matrix, allowing for a comparable analysis of the biological communities^45^ across multiple scales^44^. In comparison to other sampling methods, such as the collection of dead branching coral heads, ARMS samples may not be as representative of the coral reef fauna but provide a standardized, replicable, quantitative method.

To help predict the distribution of species, concepts such as the neutral theory and the niche theory were developed. The neutral theory states that similarity will decrease with distance given the limitation in dispersal capacities of the organisms, namely, distance decay^48^. However, the effect of environmental stochasticity in the communities present at a site may play a relevant role in coral reefs^49^. On the other hand, the niche theory explains that species similarity in communities decreases with increasing differences in environmental variables, namely environmental filtering^50^. For coral reef fishes of the Arabian Peninsula, DiBattista et al.^51^ found environmental filtering to be a cause of genetic divergence, although endemic species in the same region formed due to past isolation of the population also influence genetic diversity. Yet this has not been tested at a community level and for the cryptobiome. Conducting analysis across a broad geographic scale with varied environments will be instrumental in drawing meaningful connections between biodiversity and environmental factors^37^. This approach contributes to broader ecological insights necessary for informed environmental management and conservation efforts, namely regarding the expected trajectories of coral reefs in the Anthropocene^52,53^.

Here, we hypothesize that the communities among the four ecological regions around the Arabian Peninsula will be mainly affected by dispersal capacities, given the long geographic distance and isolation between them. Specifically, we expect a higher degree of similarity between the communities in the Gulf of Oman and the Arabian (Persian) Gulf, given their closer proximity when compared to the Red Sea. To test our hypothesis, we will examine both the role of dispersal limitation and environmental filtering in determining the community composition and diversity of the cryptobenthic communities.

## Results

Arthropoda dominated the communities of the smaller mobile fraction (106–500 µm) across all reefs and regions (Figure S-1). In the Gulf of Oman, arthropods contributed more sequences than in the other regions (Figure S-2). Annelida showed the opposite pattern in the Gulf of Oman and produced the highest proportion of reads in reef JD03 in Jeddah, in the central Red Sea (Figure S-3). The larger mobile fraction (500–2000 µm) was dominated by Annelida and Arthropoda, and in the Gulf of Oman, arthropods contributed most of the sequences (Figure S-1 and S-2). However, in Jeddah, Bryozoa was the dominant phylum in reef JD01. For the sessile fraction, Porifera contributed the greatest number of reads, except for one reef in the Gulf of Oman (BK1), one reef in the Arabian (Persian) Gulf (JK1), and one reef in the southern Red Sea (FS11), where Arthropoda dominated the communities (Figure S-1and S-4).

### Alpha diversity

**Figure 2:**
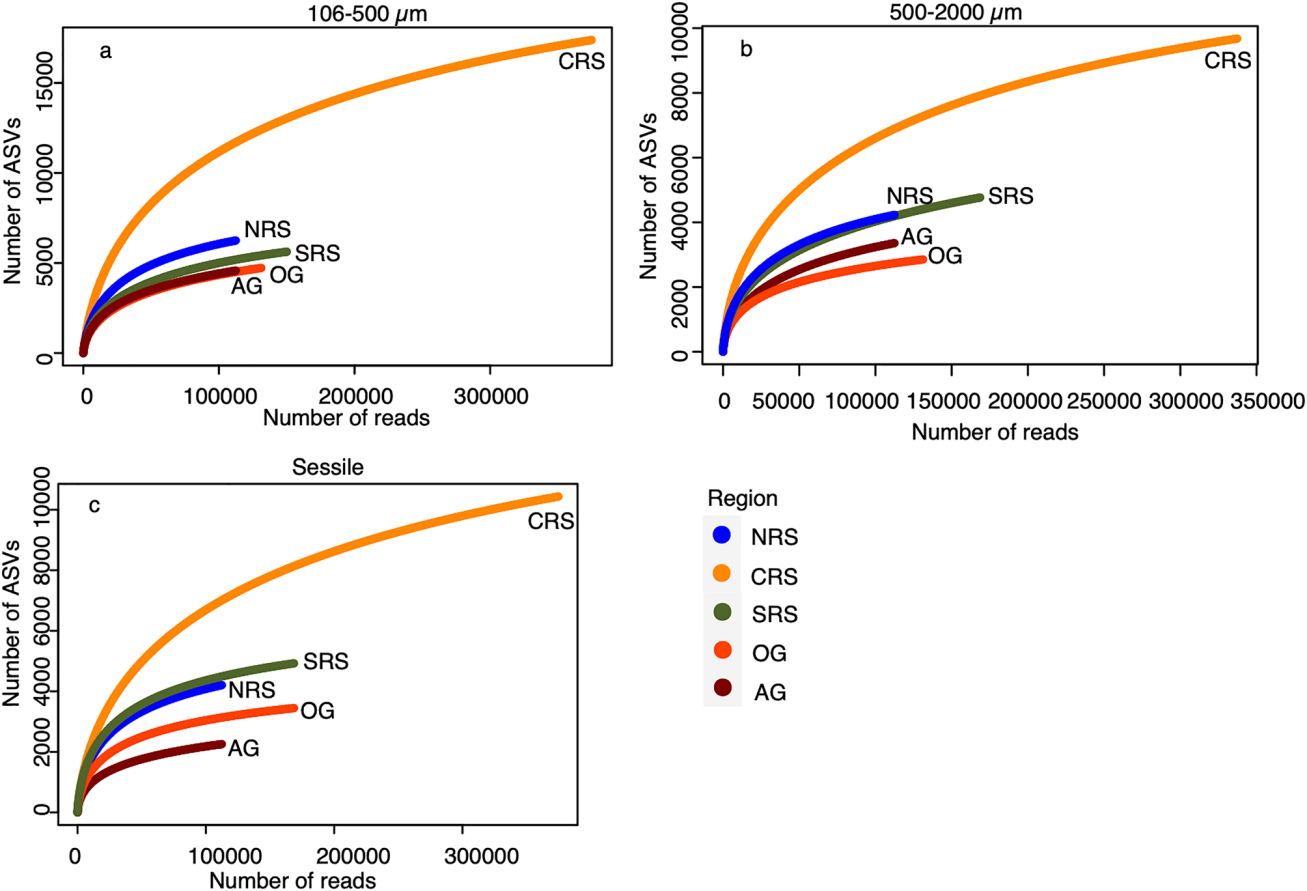
Rarefaction curves for the north (NRS), central (CRS), and south (SRS) Red Sea, and the Arabian (Persian) Gulf (AG) and Gulf of Oman (OG) for the mobile (106–500 µm and 500–2000 µm) and sessile fractions.

Rarefaction curves indicated that sampling was not sufficient in any of the regions to reveal the full diversity of the cryptobiome. All three regions of the Red Sea (north, central, and south) had a higher number of Amplicon Sequence Variants (ASVs) for an equal sequencing depth compared to the Arabian (Persian) Gulf and the Gulf of Oman, with the central Red Sea having the highest number of ASVs in all the fractions (Fig. 2). The Arabian (Persian) Gulf and the Gulf of Oman ranked differently between fractions. The larger mobile fraction (500–2000 µm) presented a higher number of ASVs in the Arabian (Persian) Gulf than in the Gulf of Oman. However, the sessile fraction presented the opposite pattern. In the smaller mobile fraction (106–500 µm), the Arabian (Persian) Gulf and the Gulf of Oman presented equivalent levels of diversity.

**Figure 3:**
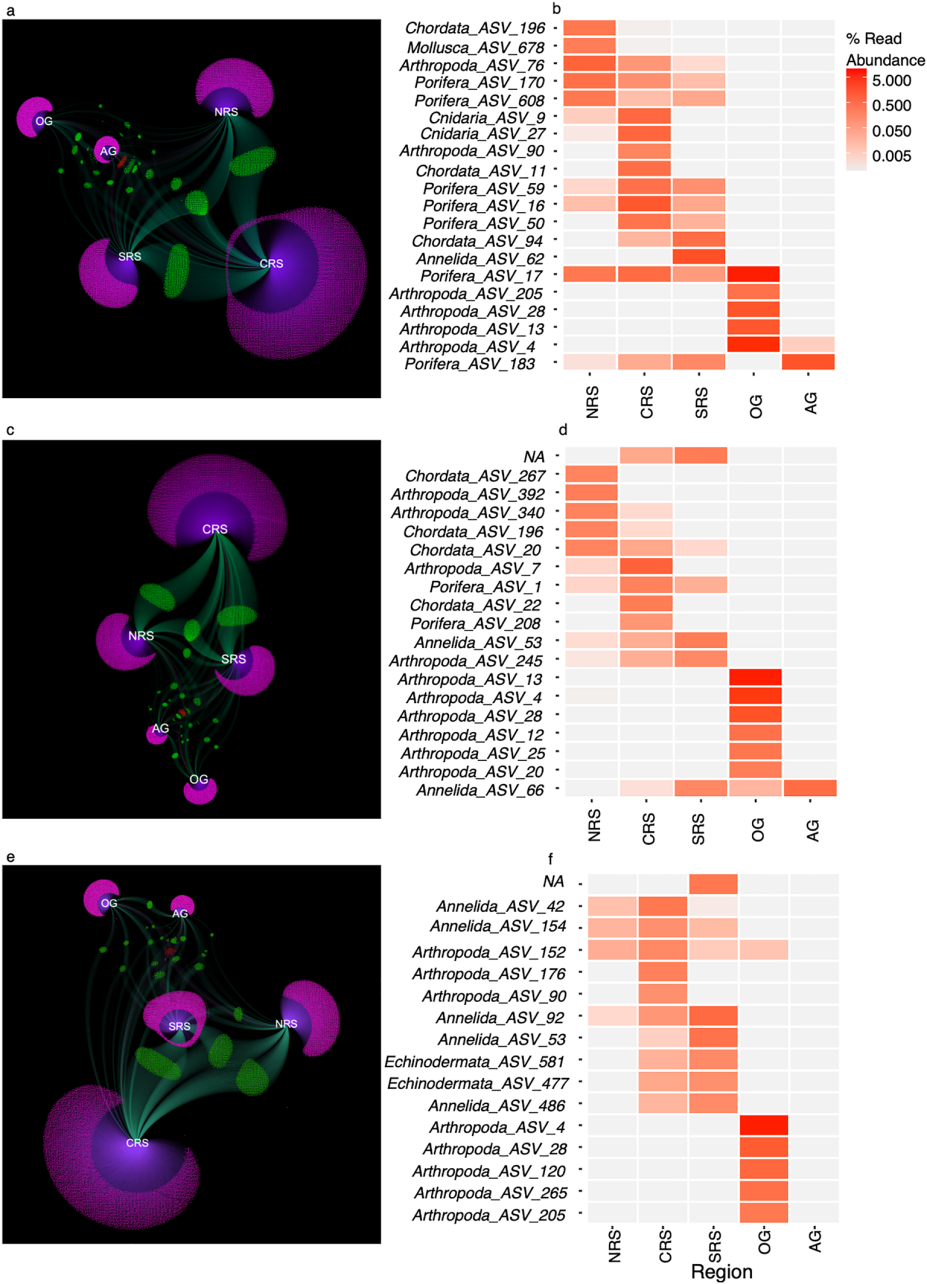
Network analysis for the shared ASVs amongst region and unique ASVs for a region for the sessile (**A**), 106–500 µm (**C**), and 500–2000 µm (**E**) fractions. Nodes for the regions were coloured blue. Purple notes represent ASVs unique to region while green ASVs indicate those that are shared between 2 and 4 regions while red nodes are ASVs shared with amongst all regions. ASVs determined as indicators as depicted for the sessile (**B**), 106–500 µm (**D**) and 500 – 2000 µm (**F**) fractions. NRS = Northern Red Sea; CRS = Central Red Sea; SRS = Southern Red Sea; OG = Gulf of Oman and AG = Arabian (Persian) Gulf. ASVs with an average contribution of less than 0.0001% were removed for visualization purposes.

Most of the ASVs were unique to a specific region, with the patterns maintained across sessile and mobile (106–500 µm and 500–2000 µm) fractions (Fig. 3). Across all regions, a small number of ASVs were shared with the majority being from the sessile fraction (46 ASVs). The lowest number of shared ASVs across all regions was in the 500–2000 µm fraction (28 ASVs). The Red Sea had more ASVs shared within its regions than with the Arabian (Persian) Gulf and the Gulf of Oman. The number of ASVs shared within the Red Sea was higher between neighboring regions than between those furthest apart (North–South). The Arabian (Persian) Gulf had more shared ASVs with regions in the Red Sea than with the Gulf of Oman. It should be, however, taken into consideration the larger sampling size of the central Red Sea.

Within the sessile fraction, 24 ASVs were identified by IndVal as indicator taxa. In the central region of the Red Sea, eight indicator taxa were present across four phyla while in the Arabian (Persian) Gulf only a single indicator taxon was present of the phylum Porifera. In the 106–500 µm, 27 indicator taxa were identified. Both the northern Red Sea and Gulf of Oman presented eight of these indicator taxa. In the northern Red Sea, these were spread across the phyla Arthropoda and Chordata, while in the Gulf of Oman, they were all taxonomically classified as Arthropoda. In the 500–2000 µm fraction, 19 indicator taxa were identified and 13 were present in the central Red Sea represented by Annelida, Arthropoda, Echinodermata, and Porifera. The Arabian (Persian) Gulf did not present indicator taxa for the 500–2000 µm.

### Community structure

Multivariate analysis indicated that there was spatial structuring with the three Red Sea regions clustering closer to each other than to either the Arabian (Persian) Gulf or Gulf of Oman (Figure S-5). In addition, the Red Sea regions also clustered with some separation among the regions. This pattern was consistent for all the fractions. In the larger mobile fraction, the northern, central, and southern Red Sea regions were separated by the vertical axis (PERMANOVA *p* < 0.001 across all fractions, Table S-1).

**Figure 4:**
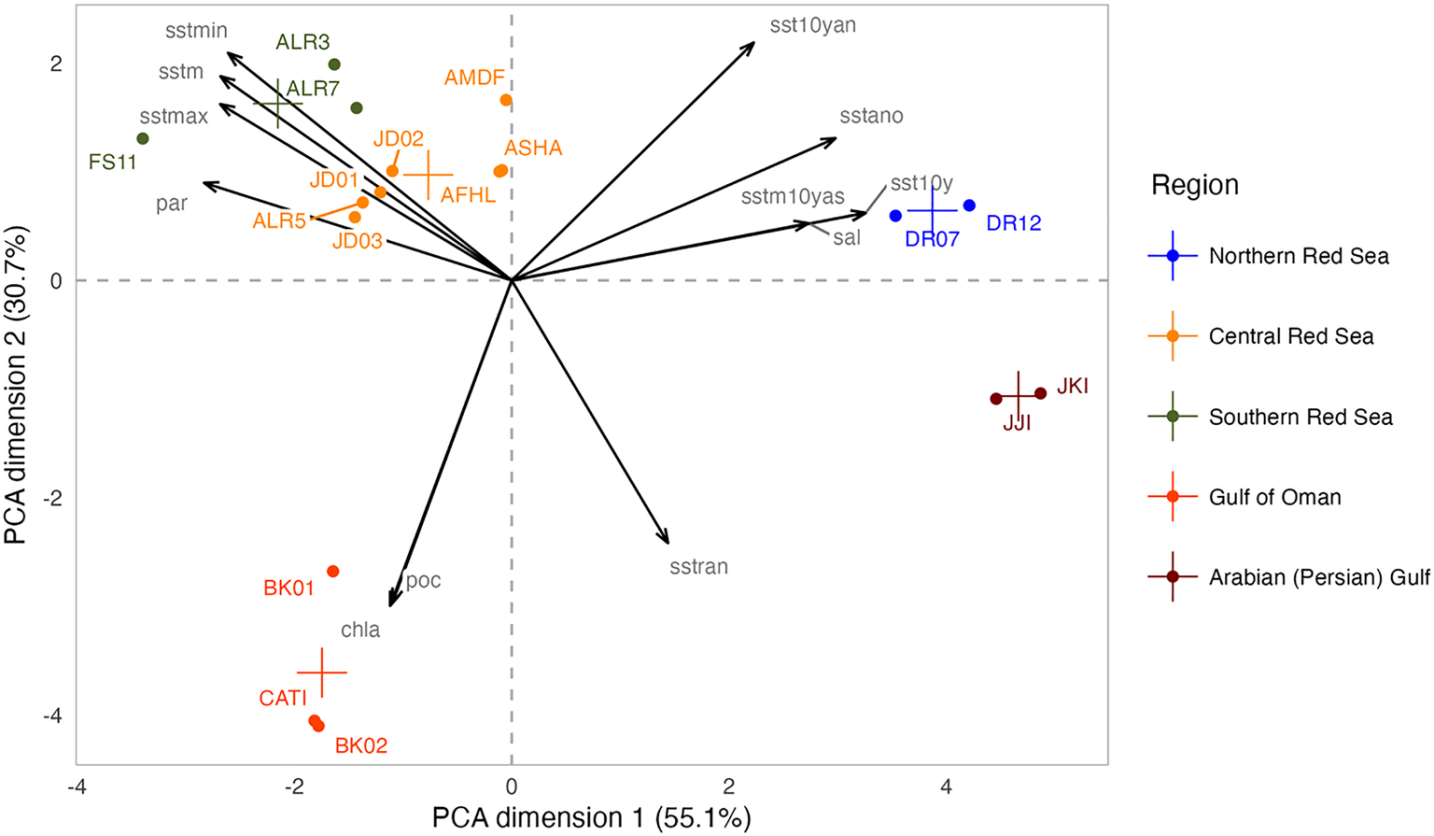
PCA biplot of the normalized environmental variables. Circles are colored according to the region of the reefs where ARMS were deployed. Sea surface temperature (SST) was computed in different ways and here presented as monthly average SST (sstm), maximum SST (sstmax), minimum SST (sstmin) and SST amplitude (sstran) were averaged for the year 2019. We also computed the annual average SST anomaly (2019; sstano); the average (2010–2020) SST anomaly (sst10y); the number of months with SST anomaly above zero (2010–2020; sst10yan) and the sum of all monthly averaged SST anomalies (2010–2020; sstm10yas). And the annual average for 2019 of particulate organic carbon (poc), chlorophyll-a (chla), and salinity (sal). The largest symbols represent the centroid for each region.

The PCA biplot of the normalized environmental variables confirmed the expected physico-chemical gradients in the Arabian Peninsula. The Gulf of Oman was associated with chlorophyll a and particulate organic carbon (POC). Salinity and the sum of all monthly averaged sea surface temperature (SST) anomalies for the period of 2010–2020 were mainly associated with reef sites in the northern Red Sea and in the Arabian (Persian) Gulf. Increasing photosynthetic active radiation (PAR) and monthly averages of SST (including minimum and maximum metrics) were associated with central and southern Red Sea regions (Fig. 4).

### Distance decay similarity with geographic and environmental distances

**Figure 5:**
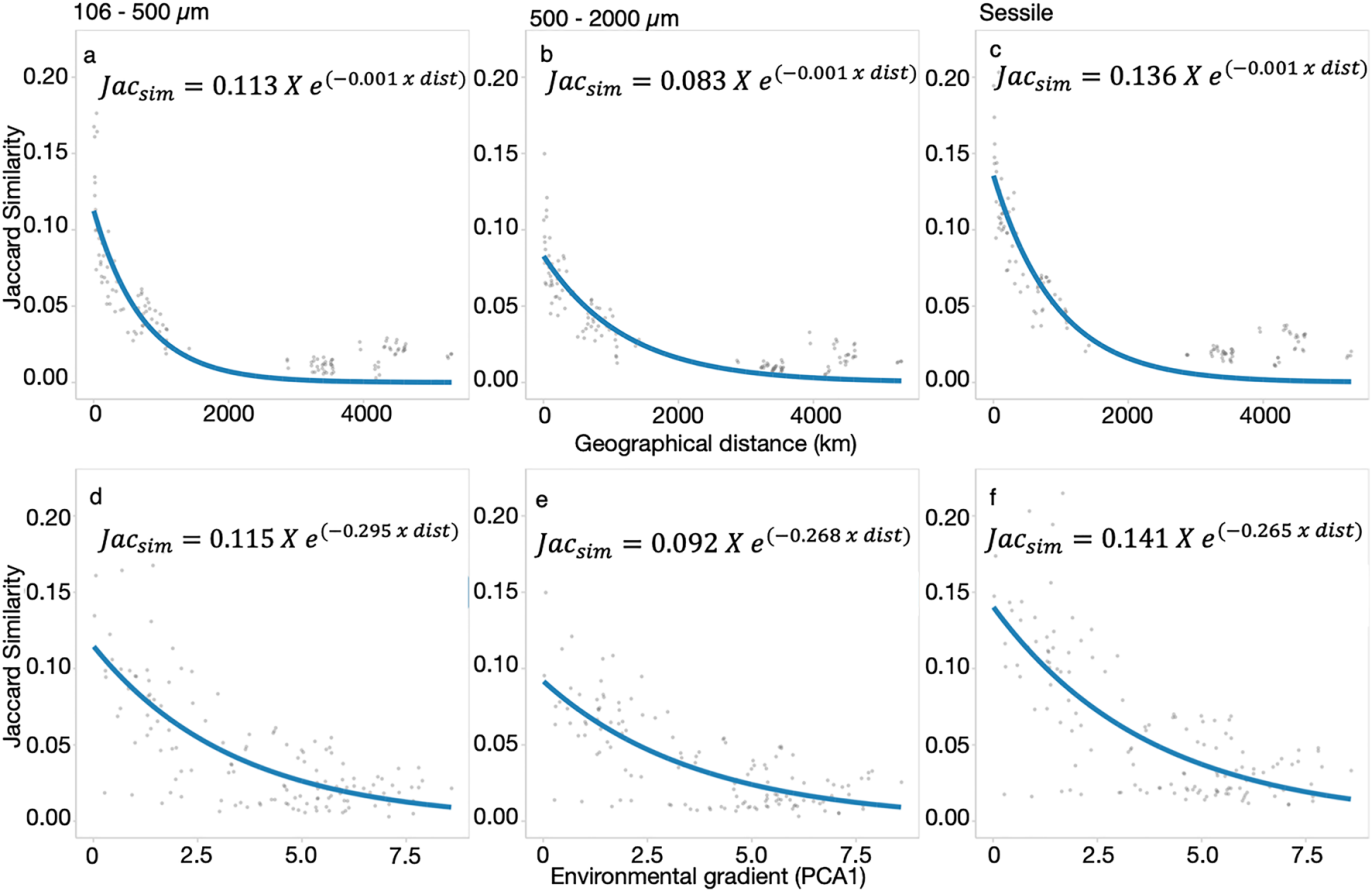
Distance decay similarity graphs using the similarity of each pair of ARMS compared against geographic distance between the ARMS for the 106–500 µm (**A**), the 500–2000 µm (**B**), and sessile (**C**) fractions. And similarity of each pair of ARMS against the environmental distance for the same pair for the 106–500 µm (**D**), the 500–2000 µm (**E**), and sessile (**F**) fractions. The similarity was obtained by subtracting the Jaccard dissimilarity of each pair of samples to 1. Environmental distance was obtained using the Euclidean distance between pair of ARMS of the two PCA dimensions that explained most of the variation of the 2019 normalized environmental parameters. A nonlinear exponential model was adapted to each graph.

Distance decay patterns, using an exponential model, indicated a rapid drop off in similarity within the first 2000 km with samples situated greater than 2000 km away from each other having low levels of similarity in community composition (Fig. 5). Likewise, similarity in community composition declined with environmental distance.

The Mantel test showed that the geographic distance and environmental distance had a significant association with similarity, with the Pearson correlation coefficient values being larger for geographic distances (Table S-2, Figure S-6). Indeed, both the Mantel test and partial Mantel tests indicated that geographic distance factors had a stronger correlation with the cryptobiome than environmental factors.

## Discussion

This study describes the cryptobiome of reefs in different regions around the Arabian Peninsula for the first time using a standardized, replicable, non-destructive approach. The results provide a baseline for future assessments targeting these fast-changing communities, particularly in comparison to corals and fish that are traditionally used in reef health assessments. Our approach characterizes these species-rich communities across a wide breadth of the biodiversity present in tropical coral reefs. Although we cannot ensure that all eukaryotic phyla have been amplified with the primer set used, we reveal that the Red Sea regions have a higher overall biodiversity than the Arabian (Persian) Gulf and the Gulf of Oman, with the central Red Sea having the highest diversity of all regions alongside the highest sample size and deployment period. Despite this bias, rarefaction curves, which are independent of the sample size, also strongly support this trend. Also, the Gulf of Oman reefs presented a lower proportion of Annelida and a higher proportion of Arthropoda sequences than other regions. The Arabian (Persian) Gulf had a similar proportion of Annelida and Arthropoda to the Red Sea samples despite its geographical proximity to the Gulf of Oman. Arthropoda and Annelida dominated the mobile fractions, in congruence with previous studies of cryptofauna using ARMS in the Red Sea^24^ and French Polynesia^45^. In terms of reads, we found a dominance of Arthropoda and Porifera in the sessile fraction, whereas previous studies in the Red Sea observed a relatively higher contribution of Chordata^24^. Pearman et al.^54^ found in sediment environmental DNA from metazoans in the central Red Sea to be dominated by Arthropoda and Annelida, congruent with our results for the mobile fractions of all sites, except one reef in the central Red Sea dominated by bryozoans. However, the sediment collections of Pearman et al.^54^ did not reflect the dominance of sponges we observed in the sessile community of the ARMS. Visual benthic surveys of the reefs studied in the Red Sea show a dominance of hard corals, macroalgae, turf algae, and soft corals^10^. The use of the sessile fraction in the ARMS favors the collection of sessile organisms of the cryptobiome, which could not be represented appropriately with sediment collection or visual reef surveys^9^.

Our study showed results consistent with the the neutral theory of ecology, highlighting the role of dispersal limitation in the distribution of species. Nevertheless, environmental filtering was partially responsible for explaining the community similarity observed between regions. Despite the wide taxonomic breadth allowed with molecular-based methods, our results seem to conform with recent meta-analyses showing an effect of dispersal limitations on the range size of marine organisms^55^.

The Red Sea is a biodiversity hotspot due to its high levels of endemism and species richness^8,31,56^. The Red Sea cryptobiome was more diverse than the other two ecological regions—Arabian (Persian) Gulf and Gulf of Oman (sensu Spalding et al.^25^). Similar patterns were highlighted by DiBattista et al.^2^ showing a higher diversity in the Red Sea for annelids, arthropods, corals, fishes, other chordates, echinoderms, and mollusks. However, the mobile fractions of the Gulf of Oman samples had a higher proportion of reads from arthropods than the Red Sea, although the opposite pattern occurred for annelids. It is possible that the more diverse coral communities of the Red Sea compared to the Arabian (Persian) Gulf and Gulf of Oman provide more niches for cryptobenthic species to colonize^8^. In addition, the Red Sea presents reef complexes across the shelf with barrier and patch reefs that shelter distinct coral communities^8^. Distinct benthic communities have been shown to influence the community that settles in barren substrates^57^. Indeed, the cryptobiome has previously been shown to be influenced by differentiations in the benthic structure^42,58–60^.

The Red Sea has been connected to the Gulf of Aden for the last 400,000 years^61^, while the Arabian (Persian) Gulf present shorelines created with the recession of polar ice sheets 3000–6000 years ago^14,62^. The younger geological age of the Arabian (Persian) Gulf (and Gulf of Oman) in comparison to the Red Sea may also contribute to the lower diversity and low levels of endemism found in both regions. The Red Sea experienced periods of isolation, facilitating speciation^31^. The Red Sea is currently in partial connection with the Gulf of Aden through the Bab al Mandeb strait allowing for Indian Ocean species tolerant to the cold nutrient rich water in the entrance to the Gulf of Aden to colonize it, adding to the pool of species inside the basin^2,31^.The Arabian (Persian) Gulf intense temperature ranges and environmental seasonality creates an intense selection pressure, filtering the species from the Indian Ocean that can establish^14^. The high selection pressure can cause speciation. However, the younger age of the Arabian (Persian) Gulf present coastlines might not be enough time for the development of species^14^. Still, Dibattista et al.^2^ found 13 percent of the Annelida species in the Arabian (Persian) Gulf to be endemic.

Overall, the central Red Sea presented the highest biodiversity and the highest number of indicator taxa. These results contrast with the patterns described for Red Sea cryptobenthic fishes, which showed higher diversity in the warmer southern reefs^20^. Latitudinal gradients in salinity and temperature, as well as the latitudinal and seasonal changes in primary production in the Red Sea are well known^4,27,28,63,64^. These environmental distinctions may have contributed to produce dissimilar reef communities between the northern, central, and southern Red Sea. Our samples from the central Red Sea were located in the southern limit of the northern Red Sea ecoregion^25^, characterized by intermediate temperatures^4^ and salinity. The central Red Sea can be a transition zone (ecotone) within the basin, which is in this case characterized by a peak in biodiversity most likely promoted by environmental conditions that can be better tolerated by organisms both from the southern and northern ecoregions^65^. Also, in the central Red Sea, ARMS were collected across a shelf gradient, which could also increase the biodiversity as ARMS were deployed in more reef types and consequently more niches to allow for the higher diversity^39^.

The 500–2000 µm fraction showed a higher diversity in the Arabian (Persian) Gulf compared to the Gulf of Oman. This pattern agrees with that described by Dibattista et al.^2^ for Annelida and Arthropoda, two phyla that are predominant in this larger size fraction of the cryptobiome. Although DiBattista et al.^2^ results are based on published information and are not directly comparable with those reached using our standard method. The opposite pattern was observed for the sessile fraction, which followed a similar pattern to that of the cryptobenthic fish in both regions^21^. Also, the Arabian (Persian) Gulf and the Gulf of Oman shared a limited number of ASVs between them and had a significant distinction in community composition. This suggests that the various fractions of the cryptobiome might be differentially affected by distinct pressures, and a better understanding of their patterns of change is needed. The Gulf of Oman has a latitudinal gradient in coral cover, which changes the benthic structure of the reefs^12^, and has been shown to influence the mobile cryptofauna^58^. In addition, the influence of the upwelling in the Arabian Sea could contribute to the distinctions observed between the Gulf of Oman and the Arabian (Persian) Gulf^34^. The Arabian Sea upwelling decrease calcification rates in corals and facilitates the development of harmful algal blooms^66,67^. It is possible that the Gulf of Oman presents a latitudinal gradient in diversity aligned with the gradient in coral cover that is not perceptible here due to the limited number of samples collected in this region. Further studies should consider exploring a latitudinal sampling in the cryptobiome for the Gulf of Oman to better understand the patterns of variability in the cryptobiome.

In the current study, geographic distance had a higher contribution to determining cryptobiome beta-diversity than environmental factors, despite both being significant. This concurs with patterns observed previously for the cryptobiome in a pan-regional study in the European Seas and the Red Sea^37^. Dispersal limitation could have a substantial impact on determining the cryptobiome community due to the potential barriers such as the Strait of Bab al Mandab, Strait of Hormuz, and the upwelling regions of the Gulf of Aden and the Arabian Sea^37^. While geographic distance had the highest influence, environmental conditions are likely to still contribute to the community patterns, as previously observed for damselfishes in the Red Sea^68^. Both the Red Sea and Arabian (Persian) Gulf have similar environmental conditions^4,5^ and they share a higher number of ASVs with each other than to the Gulf of Oman which is geographically closer and oceanographically distinct (to the Arabian (Persian) Gulf). Similar findings were reported for the cryptobiome in the low salinity Black and Baltic Seas compared to higher salinity areas (i.e., Adriatic, Mediterranean, and Atlantic Ocean) in between^37^. The results suggest that when dispersal capabilities are not limiting, niche filtering plays a relevant role in sorting the species that are able to colonize in a certain area. Indeed, distance decay plots for the environmental distance showed a deviation of some points from the exponential model that correspond to reefs in the northern Red Sea and the Arabian (Persian) Gulf. These reefs had a lower similarity than expected for their close environmental distance. Yet, they had higher similarity than expected from the model, considering the geographic distance. The coral reefs of the northern Red Sea and the Arabian (Persian) Gulf were associated with salinity and temperature variability. Both regions had higher salinity and temperature variability than their neighboring regions. This provides evidence that both limitations to dispersal through geographic distance and environmental filtering play a role in shaping the communities of the cryptobiome along the Arabian Peninsula. Congruent with our results, Brandl et al.^21^ suggested that a limiting energetic input for cryptobenthic fish communities may be a driver of change between the Arabian (Persian) Gulf and the Gulf of Oman. Saenz-Agudelo et al.^35^ found that isolation by distance and environment influence clown fishes' genetic structure more than energetic inputs. However, Dibattista et al.^2^ and Saenz-Agudelo et al.^35^ did not find the physical barriers in the Arabian Peninsula to determine species distribution or genetic diversity.

Although coral diversity and benthic community composition were not analyzed as potential environmental drivers, there is evidence that the cryptobiome is influenced by habitat type and coral diversity^58,60^. The benthic communities of the regions studied have a high contrast^9,15^, with the Arabian (Persian) Gulf harboring 66 species of corals in opposition to the 359 species in the Red Sea^8,14^. Including benthic composition in the model might have increased the influence of environmental filtering on the cryptobiome; however, data is not available across the regions. More studies are needed, particularly with more replication within the Arabian Sea, Gulf of Oman, and the Arabian (Persian) Gulf, to increase the spatial resolution and improve the model. The predominant influence of dispersal limitations in the cryptofauna communities suggests that the neutral theory of ecology acts stronger than the niche theory in this component of the reef. The ARMS remain a useful tool for studying and monitoring cryptobiome biodiversity, otherwise overlooked with traditional visual methods. Given the dispersal limitations on cryptobiome assemblages, monitoring biodiversity before and during the development of coastal areas becomes relevant to taking appropriate mitigation or restoration actions.

## Conclusions

The composition of cryptobenthic communities in different regions around the Arabian Peninsula is primarily influenced by oceanographic distance (i.e., dispersal limitation). The major influence of dispersal limitations over environmental filtering supports the neutral theory of ecology in determining the distribution of cryptobenthic communities in the region. However, the influence of both dispersal mechanisms and environmental factors underscores the complex interplay driving the ecological dynamics of these communities. The Red Sea regions are more diverse than the Arabian (Persian) Gulf and Gulf of Oman, and of particular interest in terms of biodiversity is the central Red Sea. However, reefs along the Arabian Peninsula present highly unique communities of the cryptobenthos.

## Methods

### Sampling design

We deployed ARMS in a total of 17 reefs around the Arabian Peninsula covering a total of 4 ecoregions, according to Spalding et al.^8^ (Fig. 1). Triplicate ARMS units were used at each reef except for Jeddah, where, due to weather conditions, the third ARMS was lost prior to the retrieval period. For the purpose of this study, the Northern and Central Red Sea ecoregion was further divided into 2 regions based on previous studies and considering differences in environmental and biological characteristics^10^. The Gulf of Oman and the Arabian (Persian) Gulf constituted their own region, also based on their distinct environment. Two reefs were located in the northern Red Sea, seven in the central Red Sea, three in the southern Red Sea, two in the Arabian (Persian) Gulf, and three in the Gulf of Oman.

### Deployment, recovery, and processing of ARMS

ARMS consist of nine stacked square layers of PVC of 22.5 cm separated by spaces of 1.28 cm. To mimic the coral reef matrix, the water flow is alternately restricted in the spaces between the plates. The stack of plates is placed in a base made of PVC of 45 cm by 35 cm with weights to fix the ARMS to the substrate. The base is not analyzed.

ARMS were deployed at 10 m depth between July 2016 and December 2017 and retrieved between September 2018 and February 2020. ARMS were deployed by SCUBA divers on the reefs for approximately 24 months (Table S-3). ARMS were retrieved by SCUBA divers and covered with a 106 µm mesh to retain mobile organisms. Once on the boat, the units were placed in filtered (106 µm) seawater collected and filtered on site. Back on shore, the ARMS were disassembled, and each plate brushed gently while in the filtered sea water to separate mobile from sessile organisms. This sea water was sieved through 106 µm, 500 µm, and 2000 µm gap sizes to separate the mobile organism into size classes. Only the contents from the 106–500 µm and 500–2000 µm fractions were preserved in 96% ethanol. We did not use the contents collected with the 2000 µm gap size sieve for this study. The plates were photographed on both sides. Then, the plates were scraped and the product homogenized and then preserved in 96% ethanol. Samples were then processed using metabarcoding.

### Environmental characterization

To investigate the environmental variability among reefs, the daily night-time sea surface temperature (SST) was extracted from Coral Reef Watch daily global 5 km (0.05 degree exactly) satellite coral bleaching heat stress monitoring product suite (NOAA 2000, updated twice weekly). The monthly average, maximum, minimum, and amplitude was calculated and then averaged for the year 2019. Monthly SST anomaly data was downloaded from the Coral Reef Watch website (Coral Reef Watch CoralTemp v1.0, global, 5 km resolution), and the following variables were calculated: (i) the annual average (2019); (ii) the average (2010–2020); (iii) the number of months with SST anomaly above zero (2010–2020), and iv) the sum of all monthly averaged SST anomalies (2010–2020). The annual average of chlorophyll-a concentration, particulate organic carbon, and photosynthetic active radiation was downloaded from NASA, 2021^69^. Lastly, salinity was obtained from the Copernicus website (http://marine.copernicus.eu, GLOBAL_REANALYSIS_001_030 product, approx. 8 km resolution). Values were obtained for the grid that includes the reef where ARMS were deployed. When values were not available the median of the surrounding grids was taken.

### DNA extraction, amplification, and sequencing

We used 10 g of material from each fraction (sessile, 106–500 µm, and 500–2000 µm) as input for the extractions using Powermax Soil DNA kit (MO BIO), following the manufacturer’s protocol with the exception of the bead-beating step. This step was replaced by shaking incubation overnight at 56 °C with the addition of Proteinase K (0.4 mg/mL). We amplified the DNA using a universal primer set targeting a 313 bp fragment of the COI mitochondrial gene (Forward: mlCOIintF GGWACWGGWTGAACWGTWTAYCCYCC^70^; Reverse: jgHCO2198 TAIACYTCIGGRTGICCRAARAAYCA^71^). The PCR conditions started with 3 min denaturation step at 98 °C, followed by 27 cycles at 98 °C for 10 s, 46 °C for 45 s, and 72 °C for 45 s, and a final extension step of 5 min at 72 °C. PCR reactions were conducted in triplicates using 2.5 μL of 10 × PCR Rxn Buffer, 0.5 μL of 10 mM dNTPs, 0.5 μL of 10 μM forward, 0.5 μL of 10 μM reverse primer, 1.25 μL of 50 μg L-1 BSA, 1.25 μL of 0.25 mM MgCl2, 0.25 μL of Taq DNA Polymerase, 16 μL of nuclease-free water, and 2 μL of template DNA. Triplicates were combined and 20 μl obtained for cleaning and normalizing using SequelPrep Normalization plates (ThermoFisher Scientific) obtaining a concentration of ~ 1 ng/μl. We performed a second round of PCR amplification of 8 cycles. PCR conditions consisted of 8 cycles at 95 °C for 30 s, annealing at 55 °C for 30 s, and an extension at 72 °C for 30 s. We used the Invitrogen Taq Polymerase, adding dNTPs and KAPA 2 × HiFi Hot Start ReadyMix undertaken following the manufacturer’s recommendations to add Illumina Nextera tags, followed by a second round of cleaning and normalization. We did the sequencing (2 × 300 bp) on an Illumina MiSeq sequencing platform (v3 chemistry) at the King Abdullah University of Science and Technology (KAUST) Bioscience Core Laboratory (BCL).

### Bioinformatics

We used the DADA2 package version 1.26.0^72^ within R version 4.2.2^73^ for processing of the reads after the automatically demultiplex of the sequences in the MiSeq machine. We trimmed the primers with a maximum of one mismatch allowed (parameters: −e 0.05 -discard-untrimmed). Subsequently, reads were truncated to 165 and 160 bp for forward and reverse reads respectively. We chose a maximum allowable number of “expected errors” (maxEE) of four (forward reads) and six (reverse reads) to filter the reads. Then, we dereplicated the sequences and inferred sequence variants from a parametric error matrix constructed from the first 10^8^ bp of the sequences. We discarded singletons and merged the remained reads with a minimum overlap of 10 bp with no mismatches allowed. We kept sequences with lengths from 312 to 314 bp and removed chimeric sequences using the removeBimeraDenovo script within DADA2 and pseudogenes using Multiple Alignment of Coding Sequences (MACSE^74^ against the MIDORI database^75^ as described in Leray and Knowlton^76^. We considered pseudogenes and removed sequences with a stop codon or having greater than two frame shifts. We then evenly subsampled the samples for downstream comparison (Table S-4). Taxonomy was assigned using minBoot = 51 for the assignment^77^. The R scripts can be found at https://github.com/jkpearmanbioinf/Arabian.ARMS.

### Data analysis

#### Alpha diversity

Rarefaction curves were computed using the package ‘vegan’ version 2.6–4^78^ in R to assess taxonomic diversity (using amplicon sequence variants—ASVs—as a proxy) between regions. The rarefaction was chosen given the unbalanced sampling design between regions.

To assess shared ASVs in each fraction the data was subset by size and merged by region. Shared ASVs amongst regions and ASVs unique to a region were calculated in R. To visualize the data, ASVs that contributed on average less than 0.0001% of the community were removed from the network analysis in Fig. 3 (for visualization purposes only; calculations were undertaken on the full dataset). The ASV table was converted into a graph object with the ‘igraph’ package version 1.5.1 in R and exported to the Gephi software^79^. Composition plots were created to investigate changes in the assemblage composition across regions. Networks were created using the ForceAtlas 2 algorithm with the dissuade hubs behavior included.

To assess the ASVs that were indicative of a particular region, the package ‘indicspecies’ version 1.7.14^80^ was used to identify ASVs that were significantly more abundant in a particular region for each size fraction. ASVs indicative of a region had to be significant for presence in a region (*p* < 0.05) and occur above 0.5% relative abundance in at least 10% of the samples so as to reduce the possibility of rare and low abundance ASVs being indicator taxa, which may provide an inaccurate assessment.

### Distance decay in beta diversity

The Jaccard dissimilarity distance for each pair of ARMS and for each fraction was obtained using the ‘vegan’ package. The Jaccard dissimilarity distance was deducted to 1 to obtain the Jaccard similarity distance. Distance decay plots of Jaccard similarity against the geographic and environmental distances were plotted to visualize how differences in community similarity vary with both distances and to infer on the dispersal limitations and environmental filtering driving community patterns. To obtain the geographic distance, we calculated the sea-route distance between reefs in kilometers using ‘marmap’ version 1.0.10^81^ in R. To obtain the environmental distance, we performed a PCA with the normalized environmental variables to 0 mean and 1 variance. Then, we chose the two dimensions of the PCA that explained greatest proportion of variation through a scree plot. We obtained the Euclidean distance for the two dimensions chosen, which we used as environmental distance in the ‘vegan’. We used a nonlinear model with an exponential decay formula for the geographic distance and environmental distance against the Jaccard similarity. The annual mean of 2019 of sea surface temperature, the maximum monthly temperature, the minimum monthly temperature, the monthly amplitude temperature, the temperature anomaly, the chlorophyll-a concentration, the particulate organic carbon, the salinity, and the photosynthetic active radiation, and between 2010 and 2020 the mean monthly SST anomaly, the number of months with SST anomaly above 0, and the sum of SST anomalies were used to obtain the environmental distance. We conducted a Mantel correlation between the geographic and environmental distance matrices and the Jaccard dissimilarity matrix for each fraction using the package ‘vegan’ version 2.6–4^78^. We also ran a Mantel partial correlation test to observe the independent effect of the geographic and environmental distances in community composition in the package ‘vegan’ version 2.6–4^78^.

### Supplementary Information

Supplementary Information.

## Data Availability

The datasets generated and/or analysed during the current study are available in the National Center for Biotechnology Information repository, PRJNA1063439.

## References

[1] *Status of coral reefs of the world: 2020 report. Global Coral Reef Monitoring Network (GCRMN) and International Coral Reef Initiative (ICRI).* , Souter, D., Planes, S., Wicquart, J., Logan, M., Obura, D., Staub, F., Editor. 2021.

[2] DiBattista JD (2016). A review of contemporary patterns of endemism for shallow water reef fauna in the Red Sea. J. Biogeogr..

[3] Wehe T, Fiege D (2002). Annotated checklist of the polychaete species of the seas surrounding the Arabian Peninsula: Red Sea, Gulf of Aden, Arabian Sea, Gulf of Oman, Arabian Gulf. Fauna Arabia.

[4] Berumen ML, Voolstra CR, Berumen ML (2019). The Red Sea: Environmental Gradients Shape a Natural Laboratory in a Nascent Ocean. Coral Reefs of the Red Sea.

[5] Burt JA, Feary DA, Van Lavieren H (2014). Persian Gulf reefs: An important asset for climate science in urgent need of protection. Ocean Challenge.

[6] Berumen ML (2013). The status of coral reef ecology research in the Red Sea. Coral Reefs.

[7] Vaughan GO, Burt JA (2016). The changing dynamics of coral reef science in Arabia. Mar. Pollut. Bull..

[8] Berumen ML, Voolstra CR, Berumen ML (2019). Corals of the Red Sea. Coral reefs of the Red Sea.

[9] Roberts MB (2016). Homogeneity of coral reef communities across 8 degrees of latitude in the Saudi Arabian Red Sea. Mar. Pollut. Bull..

[10] Pearman JK (2019). Disentangling the complex microbial community of coral reefs using standardized Autonomous Reef Monitoring Structures (ARMS). Mol. Ecol..

[11] Khalil MT, Bouwmeester J, Berumen ML (2017). Spatial variation in coral reef fish and benthic communities in the central Saudi Arabian Red Sea. Peerj.

[12] Burt JA (2016). Oman's coral reefs: A unique ecosystem challenged by natural and man-related stresses and in need of conservation. Mar. Pollut. Bull..

[13] Bento R (2016). The implications of recurrent disturbances within the world's hottest coral reef. Mar. Pollut. Bull..

[14] Vaughan, G.O., Al-Mansoori, N. and Burt, J.A., *The arabian gulf*, in *World seas: An environmental evaluation*, Sheppard, C., Editor. 2019, *Academic Press*. p. 1–23. DOI: 10.1016/B978-0-08-100853-9.00001-4

[15] Grizzle RE (2016). Current status of coral reefs in the United Arab Emirates: Distribution, extent, and community structure with implications for management. Mar. Pollut. Bull..

[16] Furby KA, Bouwmeester J, Berumen ML (2013). Susceptibility of central Red Sea corals during a major bleaching event. Coral Reefs.

[17] Monroe, A.A., et al., In situ observations of coral bleaching in the central Saudi Arabian Red Sea during the 2015/2016 global coral bleaching event. *Plos One*, 2018. **13**(4). DOI: 10.1371/journal.pone.0195814 10.1371/journal.pone.0195814 PMC5908266 29672556

[18] Oladi M (2021). Dynamics of *Dipsastraea pallida*-symbiont association following bleaching events across the northern Persian Gulf and Gulf of Oman. Symbiosis.

[19] Burt JA (2019). Causes and consequences of the 2017 coral bleaching event in the southern Persian/Arabian Gulf. Coral Reefs.

[20] Coker DJ (2018). Spatial patterns of cryptobenthic coral-reef fishes in the Red Sea. Coral Reefs.

[21] Brandl SJ (2020). Extreme environmental conditions reduce coral reef fish biodiversity and productivity. Nat. Commun..

[22] Pearman JK (2017). Microbial planktonic communities in the Red Sea: High levels of spatial and temporal variability shaped by nutrient availability and turbulence. Sci. Rep..

[23] Pearman JK (2016). Biodiversity patterns of plankton assemblages at the extremes of the Red Sea. F. E. M. S. Microbiol. Ecol..

[24] Carvalho S (2019). Beyond the visual: Using metabarcoding to characterize the hidden reef cryptobiome. Proc. R. Soc. B: Biol. Sci..

[25] Spalding MD (2007). Marine ecoregions of the world: A bioregionalization of coastal and shelf areas. Bioscience.

[26] Ngugi DK (2012). Biogeography of pelagic bacterioplankton across an antagonistic temperature-salinity gradient in the Red Sea. Mol. Ecol..

[27] Churchill JH (2014). The transport of nutrient-rich Indian Ocean water through the Red Sea and into coastal reef systems. J. Marine Res..

[28] Acker J (2008). Remotely-sensed chlorophyll a observations of the northern Red Sea indicate seasonal variability and influence of coastal reefs. J. Marine Syst..

[29] DeCarlo TM (2021). Patterns, drivers, and ecological implications of upwelling in coral reef habitats of the southern Red Sea. J. Geophys. Res.-Oceans.

[30] Bailey, G., The Red Sea, coastal landscapes, and Hominin dispersals, in The evolution of human populations in Arabia: Paleoenvironments, prehistory and genetics. Petraglia, M.D. and Rose, J.I., Editors. 2010, Springer Netherlands. p. 15–37. DOI: 10.1007/978-90-481-2719-1

[31] DiBattista JD (2016). On the origin of endemic species in the Red Sea. J. Biogeogr..

[32] Wiggert JD (2005). Monsoon-driven biogeochemical processes in the Arabian Sea. Prog. Oceanogr..

[33] Al-Azri AR (2010). Chlorophyll a as a measure of seasonal coupling between phytoplankton and the monsoon periods in the Gulf of Oman. Aqu. Ecol..

[34] Coles SL (1997). Reef corals occurring in a highly fluctuating temperature environment at Fahal Island, Gulf of Oman (Indian Ocean). Coral Reefs.

[35] Saenz-Agudelo P (2015). Seascape genetics along environmental gradients in the Arabian Peninsula: Insights from ddRAD sequencing of anemonefishes. Molecular Ecology.

[36] Reaka-Kudla, M.L., et al., *The global biodiversity of coral reefs: A comparison with rain forests*. in *Biodiversity II: Understanding and protecting our biological resources,* 1997,* Joseph Henry Press*. **2**: p. 551. DOI: 10.17226/4901

[37] Pearman JK (2020). Pan-regional marine benthic cryptobiome biodiversity patterns revealed by metabarcoding Autonomous Reef Monitoring Structures. Mol. Ecol..

[38] Pearman JK (2016). Please mind the gap - Visual census and cryptic biodiversity assessment at central Red Sea coral reefs. Marine Environ. Res..

[39] Pearman JK (2018). Cross-shelf investigation of coral reef cryptic benthic organisms reveals diversity patterns of the hidden majority. Sci. Rep..

[40] Al-Rshaidat MMD (2016). Deep COI sequencing of standardized benthic samples unveils overlooked diversity of Jordanian coral reefs in the northern Red Sea. Genome.

[41] Conradi M (2018). Five new coexisting species of copepod crustaceans of the genus *Spaniomolgus* (Poecilostomatoida: Rhynchomolgidae), symbionts of the stony coral *Stylophora pistillata* (Scleractinia). Zookeys.

[42] Troyer EM, Coker DJ, Berumen ML (2018). Comparison of cryptobenthic reef fish communities among microhabitats in the Red Sea. Peerj.

[43] Kandler NM (2019). Hyperdiverse macrofauna communities associated with a common sponge, Stylissa carteri, shift across ecological gradients in the central Red Sea. Diversity.

[44] Villalobos R (2022). Inter-annual variability patterns of reef cryptobiota in the central Red Sea across a shelf gradient. Scientific Reports.

[45] Ransome E (2017). The importance of standardization for biodiversity comparisons: A case study using Autonomous Reef Monitoring Structures (ARMS) and metabarcoding to measure cryptic diversity on Mo'orea coral reefs, French Polynesia. Plos One.

[46] Wolfe K, Mumby PJ (2020). RUbble biodiversity samplers: 3D-printed coral models to standardize biodiversity censuses. Methods Ecol. Evol..

[47] Johnson MD, Price NN, Smith JE (2022). Calcification accretion units (CAUs): A standardized approach for quantifying recruitment and calcium carbonate accretion in marine habitats. Methods Ecol. Evol..

[48] Hubbell, S.P. *The unified neutral theory of biodiversity and biogeography*, in *Monographs in Population Biology. *2001. p. i-xiv, 1–375.

[49] Dornelas M, Connolly SR, Hughes TP (2006). Coral reef diversity refutes the neutral theory of biodiversity. Nature.

[50] Thakur MP (2020). Towards an integrative understanding of soil biodiversity. Biol. Rev..

[51] DiBattista JD (2020). Population genomic response to geographic gradients by widespread and endemic fishes of the Arabian Peninsula. Ecol. Evol..

[52] Camp EF (2018). The future of coral reefs subject to rapid climate change: Lessons from natural extreme environments. Front. Marine Sci..

[53] Hughes TP (2017). Coral reefs in the Anthropocene. Nature.

[54] Pearman JK, Irigoien X, Carvalho S (2016). Extracellular DNA amplicon sequencing reveals high levels of benthic eukaryotic diversity in the central Red Sea. Marine Genom..

[55] Alzate A, Onstein RE (2022). Understanding the relationship between dispersal and range size. Ecol. Lett..

[56] Bowen BW (2013). The origins of tropical marine biodiversity. Trends Ecol. Evol..

[57] Roth F (2018). Coral reef degradation affects the potential for reef recovery after disturbance. Marine Environ. Res..

[58] Enochs IC, Manzello DP (2012). Species richness of motile cryptofauna across a gradient of reef framework erosion. Coral Reefs.

[59] Head CEI (2015). High prevalence of obligate coral-dwelling decapods on dead corals in the Chagos Archipelago, central Indian Ocean. Coral Reefs.

[60] Ip YCA (2023). Seq' and ARMS shall find: DNA (meta)barcoding of Autonomous Reef Monitoring Structures across the tree of life uncovers hidden cryptobiome of tropical urban coral reefs. Mol. Ecol..

[61] Lambeck K (2011). Sea level and shoreline reconstructions for the Red Sea: Isostatic and tectonic considerations and implications for hominin migration out of Africa. Quaternary Sci. Rev..

[62] Kassler, P., *The structural and geomorphic evolution of the Persian Gulf*, in *The Persian Gulf*. 1973, *Springer*. p. 11–32. 10.1007/978-3-642-65545-6

[63] Raitsos DE (2013). Remote sensing the phytoplankton seasonal succession of the Red Sea. Plos One.

[64] Wafar M (2016). Patterns of distribution of inorganic nutrients in Red Sea and their implications to primary production. J. Marine Syst..

[65] Smith TB (1997). A role for ecotones in generating rainforest biodiversity. Science.

[66] Harrison PJ, Piontkovski S, Al-Hashmi K (2017). Understanding how physical-biological coupling influences harmful algal blooms, low oxygen and fish kills in the Sea of Oman and the Western Arabian Sea. Marine Pollut. Bull..

[67] Spreter PM (2022). Calcification response of reef corals to seasonal upwelling in the northern Arabian Sea (Masirah Island, Oman). Biogeosciences.

[68] Robitzch VSN (2016). Productivity and sea surface temperature are correlated with the pelagic larval duration of damselfishes in the Red Sea. Marine Pollut. Bull..

[69] NASA, 2021. NASA Oceancolor website, retrieved on the 13th of April 2021 from https://oceandata.sci.gsfc.nasa.gov/opendap/catalog.xml (MODIS Aqua; 4 km resolution).

[70] Leray M (2013). A new versatile primer set targeting a short fragment of the mitochondrial COI region for metabarcoding metazoan diversity: Application for characterizing coral reef fish gut contents. Front. Zool..

[71] Geller J (2013). Redesign of PCR primers for mitochondrial cytochrome c oxidase subunit I for marine invertebrates and application in all-taxa biotic surveys. Mol. Ecol. Resour..

[72] Callahan BJ (2016). DADA2: High-resolution sample inference from Illumina amplicon data. Nat. Methods.

[73] Team, R.C. *A Language and Environment for Statistical Computing*. 2020: R Foundation for Statistical Computing.

[74] Ranwez V (2011). MACSE: Multiple Alignment of Coding SEquences accounting for frameshifts and stop codons. Plos One.

[75] Machida RJ (2017). Data descriptor: Metazoan mitochondrial gene sequence reference datasets for taxonomic assignment of environmental samples. Sci. Data.

[76] Leray M, Knowlton N (2015). DNA barcoding and metabarcoding of standardized samples reveal patterns of marine benthic diversity. Proc. Natl. Acad. Sci. USA.

[77] Wang Q (2007). Naive Bayesian classifier for rapid assignment of rRNA sequences into the new bacterial taxonomy. Appl. Environ. Microbiol..

[78] Oksanen, J.F., et al., *vegan: Community Ecology Package*. 2018, R package version 2.5–1. https://CRAN.R-project.org/package=vegan.

[79] Bastian, M., Heymann, S., and Jacomy, M., *Gephi: An open source software for exploring and manipulating networks*. in *Proceedings of the international AAAI conference on web and social media*. 2009. https://igraph.org/

[80] De Caceres M, Jansen F (2016). Package ‘indicspecies’. Indicators.

[81] Pante E, Simon-Bouhet B (2013). marmap: A package for importing, plotting and analyzing bathymetric and topographic data in R. Plos One.

