# Supplementary material for: Biodiversity patterns of the coral reef cryptobiota around the Arabian Peninsula
Source: Sci Rep. 2024 Apr 25;14:9532. doi: 10.1038/s41598-024-60336-8 (PMC11045746; doi:10.1038/s41598-024-60336-8)
Supplement: Supplementary file 1 — Supplementary Information. [file 41598_2024_60336_MOESM1_ESM.pdf]

Biodiversity patterns of the coral reef cryptobiota around the Arabian Peninsula

Rodrigo Villalobos[1], Eva Aylagas[1], John K. Pearman[1,2], Joao Curdia[1], Darren Coker[1], Alyssa Clothilde Bell[1,3], Shannon D. Brown[1,4], Katherine Rowe[1,5], Diego Lozano-Cortés[6], Lotfi J. Rabaoui[7,8], Alyssa Marshell[9.10], Mohammad Qurban[7,11], Burton Jones[1], Michael Lee Berumen[1], & Susana Carvalho[1*]

1. King Abdullah University of Science and Technology (KAUST), Red Sea Research Center, Thuwal 23955-6900, Saudi Arabia
2. Coastal and Freshwater Group, Cawthron Institute, Nelson, New Zealand
3. Department of Biology, University of Konstanz, Konstanz, Germany
4. Cooperative Institute for Climate, Ocean, and Ecosystem Studies, University of Washington, Seattle, WA, United States
5. School of Science, The University of Waikato, Hamilton, New Zealand
6. Environmental Protection, Saudi Aramco, Dhahran, Saudi Arabia
7. Center for Environment & Marine Studies, Research Institute, King Fahd University of Petroleum & Minerals, Dhahran 31261, Eastern Province, Kingdom of Saudi Arabia
8. National Center for Wildlife, Riyadh, Saudi Arabia
9. Sultan Qaboos University, Al Seeb Al Khoudh SQU SEPS Muscat OM, 123, Oman
10. Institute for Marine and Antarctic Studies, University of Tasmania, Hobart, Tasmania, 7053
11. Ministry of Environment, Water and Agriculture, Kingdom of Saudi Arabia

*Corresponding author susana.carvalho@kaust.edu.sa

Supplementary material

Table S-1. Results from the mobile fractions 106-500 µm and 500-2000 µm and the sessile fraction of the post hoc analysis of the permutational multivariate analysis of variance in the Jaccard dissimilarity matrices.

		P values				R2			
Fraction	Region	AG	CRS	NRS	OG	AG	CRS	NRS	OG
500-2000	CRS	0.01	-	-	-	0.07	-	-	-
	NRS	0.02	0.01	-	-	0.15	0.07	-	-
	OG	0.03	0.01	0.02	-	0.17	0.09	0.18	-
	SRS	0.01	0.01	0.01	0.01	0.11	0.06	0.11	0.14
106-500	CRS	0.01	-	-	-	0.09	-	-	-
	NRS	0.04	0.01	-	-	0.20	0.08	-	-
	OG	0.01	0.01	0.01	-	0.24	0.12	0.24	-
	SRS	0.05	0.01	0.02	0.01	0.15	0.06	0.15	0.19
Sessile	CRS	0.01	-	-	-	0.13	-	-	-
	NRS	0.01	0.01	-	-	0.22	0.08	-	-
	OG	0.01	0.01	0.03	-	0.22	0.13	0.19	-
	SRS	0.01	0.01	0.01	0.01	0.17	0.07	0.12	0.16

Table S-2. Mantel correlations, Mantel partial correlation, and Multiple Regression on distance Matrices (MRM) between the geographic and Environmental distance matrices and the Jaccard dissimilarity matrix for each fraction. Geo. = Geographic distance, Env. = Environmental distance, P value = probability value, 106 µm = 106-500 µm fraction, 500 µm = 500-2000 µm fraction, Sessile = Sessile fraction, R = Mantel statistic using the Pearson correlation, R^2 = regression R-squared

	**Mantel Correlation**					
	**Geographical**		**Environmental**			
	**R**	**P value**	**R**	**P value**		
106-500 µm	0.93	0.0001	0.77	0.0001		
500-2000 µm	0.92	0.0001	0.76	0.0001		
Sessile	0.94	0.0001	0.74	0.0001		
	**Mantel Partial Correlation**				**MRM**	
	**Geo. (-Env.)**		**Env. (-Geo.)**		**Geo. + Env.**	
	**R**	**P value**	**R**	**P value**	**R^2**	**P value**
106-500 µm	0.83	0.0001	0.21	0.0140	0.87	0.0010
500-2000 µm	0.80	0.0001	0.20	0.0194	0.85	0.0010
Sessile	0.85	0.0001	0.09	0.1466	0.88	0.0010

Table S-3. Metadata for reefs where ARMS were retrieved.

Region	Site	Latitude N	Longitude E	Deployed	Retrieval Date
North Red Sea	DR12	27.681	35.442	July 2016	September 2018
North Red Sea	DR07	27.273	35.642	July 2016	September 2018
Central Red Sea	ASHA	22.298	39.046	July 2017	May 2019
Central Red Sea	AFHL	22.228	38.965	July 2017	June 2019
Central Red Sea	AMDF	22.089	38.778	July 2017	November 2019
Central Red Sea	JD01	21.453	39.112	April 2017	June 2019
Central Red Sea	JD02	21.225	39.121	August 2017	June 2019
Central Red Sea	JD03	21.082	39.201	April 2017	June 2019
Central Red Sea	ALR5	20.496	39.636	May 2017	May 2019
South Red Sea	ALR7	20.122	40.218	May 2017	May 2019
South Red Sea	ALR3	19.907	40.523	May 2017	May 2019
South Red Sea	FS11	16.895	42.397	November 2017	October 2019
Oman Gulf	CAT	23.586	58.610	November 2016	March 2019
Oman Gulf	BK1	23.512	58.760	November 2016	March 2019

Oman Gulf	BK2	23.527	58.740	November 2016	March 2019
Arabian Gulf	JKI	27.719	49.838	December 2017	February 2020
Arabian Gulf	JJI	27.360	49.889	December 2017	February 2020

Table S-4. Number of reads kept at each step for each ARMS sample.

Reef	#	Sample	input	filtered	denoisedF	denoisedR	merged	final
JJI	1	JJI-ARMS1-100	144092	127336	125992	125485	124153	109259
JJI	1	JJI-ARMS1-500	170102	150970	148177	147724	146478	136769
JJI	1	JJI-ARMS1-Sessile	166349	147018	142320	141832	140438	125659
JJI	2	JJI-ARMS2-100	163122	144298	141382	140764	139251	113308
JJI	2	JJI-ARMS2-500	150197	133195	130784	130424	129056	114176
JJI	2	JJI-ARMS2-Sessile	145485	128414	123169	122991	121792	97599
JJI	3	JJI-ARMS3-100	161599	143475	142486	141777	140881	121299
JJI	3	JJI-ARMS3-500	156443	138519	135117	134555	132457	112420
JJI	3	JJI-ARMS3-Sessile	158386	139921	136258	135954	133959	121134
JKI	1	JKI-ARMS1-100	178084	157364	150859	150046	147747	136294
JKI	1	JKI-ARMS1-500	184259	162896	159998	159315	158332	140377
JKI	1	JKI-ARMS1-Sessile	162501	143856	141939	141720	140821	128210
JKI	2	JKI-ARMS2-100	161296	142536	134072	133120	130976	115350
JKI	2	JKI-ARMS2-500	181171	160954	157685	157263	155652	150058
JKI	2	JKI-ARMS2-Sessile	165805	147029	143444	142982	141131	134458
JKI	3	JKI-ARMS3-100	169284	149615	148047	147311	145574	133613
JKI	3	JKI-ARMS3-500	157939	140055	138425	138188	137250	124437
JKI	3	JKI-ARMS3-Sessile	178016	157580	155843	155456	153325	138074
BK1	1	BK1-ARMS1-100	321463	308945	301159	298272	292930	270769
BK1	1	BK1-ARMS1-Sessile	306119	294259	283039	281912	279431	264623
BK1	2	BK1-ARMS2-100	300220	288582	283035	282069	279231	232894
BK1	2	BK1-ARMS2-200	325376	310455	278707	277538	273379	253359
BK1	2	BK1-ARMS2-Sessile	267541	257528	249058	248017	246437	228546
BK1	3	BK1-ARMS3-100	278298	265461	236601	234520	233219	189425
BK1	3	BK1-ARMS3-200	181248	173599	154774	154080	151489	117647
BK1	3	BK1-ARMS3-Sessile	264589	253948	248671	248005	246698	234038
BK2	1	BK2-ARMS1-100	296585	284133	276615	274490	269806	245274
BK2	1	BK2-ARMS1-500	260432	250651	245003	244629	239755	219767
BK2	1	BK2-ARMS1-Sessile	259750	249220	237758	236664	233125	215352
BK2	2	BK2-ARMS2-100	271973	260962	252244	249978	246590	221794
BK2	2	BK2-ARMS2-500	228336	220870	216571	215972	213249	183373

BK2	2	BK2-ARMS2-Sessile	216211	207043	202101	201348	198779	188095
BK2	3	BK2-ARMS3-100	272158	260894	256258	253865	250284	230216
BK2	3	BK2-ARMS3-500	258183	247557	232865	232170	231305	215371
BK2	3	BK2-ARMS3-Sessile	26730	24251	23526	22513	21991	20510
CAT	1	CAT1-ARMS1-100	255839	246783	243603	242093	239312	207032
CAT	1	CAT1-ARMS1-500	168898	163512	160589	160238	158812	139569
CAT	1	CAT1-ARMS1-Sessile	291357	279349	270459	267691	265328	253665
CAT	2	CAT1-ARMS2-Sessile	235961	226760	215839	213321	206966	194671
CAT	3	CAT1-ARMS3-100	323445	310258	305865	302768	298715	256711
CAT	3	CAT1-ARMS3-500	337637	325026	322184	322025	317153	270318
CAT	3	CAT1-ARMS3-Sessile	252753	243447	234234	233338	231897	216216
DR07	1	DR07-ARMS1-100	115419	106944	103362	104674	99079	91389
DR07	1	DR07-ARMS1-500	118536	109027	107130	107589	84034	79851
DR07	2	DR07-ARMS2-100	241119	221889	213683	219046	162179	150323
DR07	2	DR07-ARMS2-500	197168	184276	178960	181584	143529	134925
DR07	3	DR07-ARMS3-100	101279	93156	89102	91214	83416	75017
DR07	3	DR07-ARMS3-500	84624	78287	74682	77297	68656	59451
DR07	1	DR07-ARMS1-Sessile	174957	167442	163524	165867	160862	149123
DR07	2	DR07-ARMS2-Sessile	177994	167678	154553	165225	141994	126119
DR07	3	DR07-ARMS3-Sessile	156469	150249	144290	148301	140879	128274
DR12	1	DR12-ARMS1-100	283730	262126	249659	258379	194109	157997
DR12	1	DR12-ARMS1-500	143754	126129	119130	123295	115561	101849
DR12	1	DR12-ARMS1-Sessile	151015	139919	136276	138011	134080	116158
DR12	2	DR12-ARMS2-100	260507	234361	209848	229085	175684	154070
DR12	2	DR12-ARMS2-500	149616	140480	134033	138840	123943	112908
DR12	2	DR12-ARMS2-Sessile	178551	162725	151470	159978	147382	131020
DR12	3	DR12-ARMS3-100	299673	280108	267972	274153	257148	225351
DR12	3	DR12-ARMS3-500	260739	243920	232866	241063	224733	208546
DR12	3	DR12-ARMS3-Sessile	174443	159013	147308	156675	145067	134041
AFHL	1	AFHL-ARMS1-100	162489	156265	149650	153645	135016	119913
AFHL	1	AFHL-ARMS1-500	177814	172420	161468	170300	158311	140320
AFHL	1	AFHL-ARMS1-Sessile	127478	120565	110984	119270	109014	100391
AFHL	2	AFHL-ARMS2-100	148077	143849	140445	141399	119508	107758
AFHL	2	AFHL-ARMS2-500	59096	56513	52261	55135	50096	46830
AFHL	2	AFHL-ARMS2-Sessile	178024	172375	163972	171221	161294	150758
AFHL	3	AFHL-ARMS3-100	72679	70943	69872	69565	66778	62133
AFHL	3	AFHL-ARMS3-500	1395	1343	1280	1278	1159	1032
AFHL	3	AFHL-ARMS3-Sessile	146260	129871	127364	127593	124119	112559
ALR3	1	ALR3-ARMS1-100	184458	168450	139112	166787	134720	99741

ALR3	1	ALR3-ARMS1-500	163440	157353	136166	156022	134139	117736
ALR3	1	ALR3-ARMS1-Sessile	197818	191578	180738	190590	178994	163139
ALR3	2	ALR3-ARMS2-100	184771	165088	129385	163923	121097	57028
ALR3	2	ALR3-ARMS2-500	143281	138944	118242	137860	116069	95805
ALR3	2	ALR3-ARMS2-Sessile	156659	152540	143684	151416	142135	105261
ALR3	3	ALR3-ARMS3-100	204188	178162	139653	176427	135352	82650
ALR3	3	ALR3-ARMS3-500	67357	65948	62636	65108	61076	53248
ALR3	3	ALR3-ARMS3-Sessile	176208	171671	161578	171071	160634	140009
ALR5	1	ALR5-ARMS1-100	120049	116137	109918	113850	106468	100113
ALR5	1	ALR5-ARMS1-500	105930	102871	95595	101996	93975	85262
ALR5	1	ALR5-ARMS1-Sessile	154728	147426	141205	146042	139999	134436
ALR5	2	ALR5-ARMS2-100	108795	106081	100477	104378	97012	89142
ALR5	2	ALR5-ARMS2-500	71182	69502	67330	68521	65997	58522
ALR5	2	ALR5-ARMS2-Sessile	156111	151524	142731	150875	141639	134086
ALR5	3	ALR5-ARMS3-100	106940	105274	104933	104995	104613	100395
ALR5	3	ALR5-ARMS3-500	128279	123923	119768	123379	119253	109146
ALR5	3	ALR5-ARMS3-Sessile-I	174715	154834	147865	152050	145210	126204
ALR7	1	ALR7-ARMS1-100	86416	82149	73289	79958	66387	58808
ALR7	1	ALR7-ARMS1-500	213287	206762	188167	204490	182653	171183
ALR7	1	ALR7-ARMS1-Sessile	96108	93061	88508	92086	87141	78853
ALR7	2	ALR7-ARMS2-100	103846	100084	89646	98534	83971	73923
ALR7	2	ALR7-ARMS2-500	158802	155746	148386	154544	141727	135306
ALR7	2	ALR7-ARMS2-Sessile	96509	93448	88409	92496	87091	78904
ALR7	3	ALR7-ARMS3-100	24	16	8	7	4	4
ALR7	3	ALR7-ARMS3-500	146830	144022	132731	142438	128953	118027
ALR7	3	ALR7-ARMS3-Sessile	50707	49067	47202	48199	46506	43317
ASHA	1	ASHO-ARMS1-100	195438	189352	177070	187041	151016	134823
ASHA	1	ASHO-ARMS1-500	166188	161430	151603	159948	149361	139327
ASHA	1	ASHO-ARMS1-Sessile	242396	233190	212608	230721	209440	194498
ASHA	2	ASHO-ARMS2-100	195661	190455	176066	187818	168426	151755
ASHA	2	ASHO-ARMS2-500	124530	121270	99612	119958	96887	91802
ASHA	2	ASHO-ARMS2-Sessile	178435	174367	171963	173325	170300	155290
ASHA	3	ASHO-ARMS3-100	173273	167448	157482	164900	152999	136203
ASHA	3	ASHO-ARMS3-500	70771	69140	62781	68208	60762	57885
ASHA	3	ASHO-ARMS3-Sessile	184153	181127	175518	180299	173953	160321
JD01	1	JD01-ARMS1-100	149325	141857	137003	140168	129410	117620

JD01	1	JD01-ARMS1-500	38994	36108	34991	35342	34242	31079
JD01	1	JD01-ARMS1-Sessile	169360	164177	152820	162459	150771	139487
JD01	2	JD01-ARMS2-100	182108	175781	169361	173095	167607	151142
JD01	2	JD01-ARMS2-500	188788	182899	168973	180318	154714	138206
JD01	2	JD01-ARMS2-Sessile	179749	172099	160719	169508	159186	149199
JD01	3	JD01-ARMS3-100	185814	180481	174749	178878	171213	156379
JD01	3	JD01-ARMS3-500	165226	160341	153907	159274	152754	133617
JD01	3	JD01-ARMS3-Sessile	157474	139632	125256	134063	123734	118050
JD02	2	JD02-ARMS2-100	69748	67728	62562	65213	60171	54653
JD02	2	JD02-ARMS2-500	107	70	34	22	16	12
JD02	2	JD02-ARMS2-Sessile	142907	137558	129098	136038	127842	121978
JD02	3	JD02-ARMS3-100	134057	127173	119774	121188	99447	90533
JD02	3	JD02-ARMS3-500	167199	155699	141655	149915	136287	125095
JD02	3	JD02-ARMS3-Sessile	126056	122031	116111	120127	113967	105119
JD03	1	JD03-ARMS1-100	145876	138898	129220	137166	122592	109223
JD03	1	JD03-ARMS1-500	130312	125500	114033	124291	111985	101149
JD03	1	JD03-ARMS1-Sessile	140393	130685	125290	129877	124062	115000
JD03	2	JD03-ARMS2-100	182173	175789	167756	173285	162327	145382
JD03	2	JD03-ARMS2-500	162092	155135	143298	152944	140258	131795
JD03	2	JD03-ARMS2-Sessile	190394	181851	176877	179174	173972	145224
JD03	3	JD03-ARMS3-100	131339	124204	116915	121854	111943	99648
JD03	3	JD03-ARMS3-500	92476	88656	84363	87372	79986	72761
JD03	3	JD03-ARMS3-Sessile	195219	188589	180171	186836	177433	166031
ALR5	3	ALR5-ARMS3-Sessile-II	163618	144233	127721	128231	127198	119179
AMDF	1	AMF-ARMS1-100	170671	150561	143463	143446	139833	126245
AMDF	1	AMF-ARMS1-500	165724	146038	140911	141936	138027	124420
AMDF	1	AMF-ARMS1-Sessile	99900	88530	81218	86197	78903	72555
AMDF	2	AMF-ARMS2-100	156728	137364	127081	133297	123803	111510
AMDF	2	AMF-ARMS2-500	155466	137652	129218	134467	126625	112245
AMDF	2	AMF-ARMS2-Sessile	88135	76741	73366	73579	71436	60869
AMDF	3	AMF-ARMS3-100	170902	150454	139458	146152	135711	121420
AMDF	3	AMF-ARMS3-500	166194	147462	140903	144803	138810	131925
AMDF	3	AMF-ARMS3-Sessile	119319	105138	96256	99790	94003	82715
FS11	1	FS11-ARMS1-100	127467	112643	103771	106635	101396	86107
FS11	1	FS11-ARMS1-500	132833	117583	107485	112276	105234	95498
FS11	1	FS11-ARMS1-Sessile	157354	139827	133112	134995	130606	121149
FS11	2	FS11-ARMS2-100	128742	114183	108946	108444	105901	100286
FS11	2	FS11-ARMS2-500	171291	151817	146731	147269	143049	132987

FS11	2	FS11-ARMS2-Sessile	142570	126228	124402	125400	123275	112293
FS11	3	FS11-ARMS3-100	127194	112579	104960	104281	102502	89846
FS11	3	FS11-ARMS3-500	147227	130836	125378	128001	122911	108598
FS11	3	FS11-ARMS3-Sessile	166583	147692	138037	146637	136887	119950

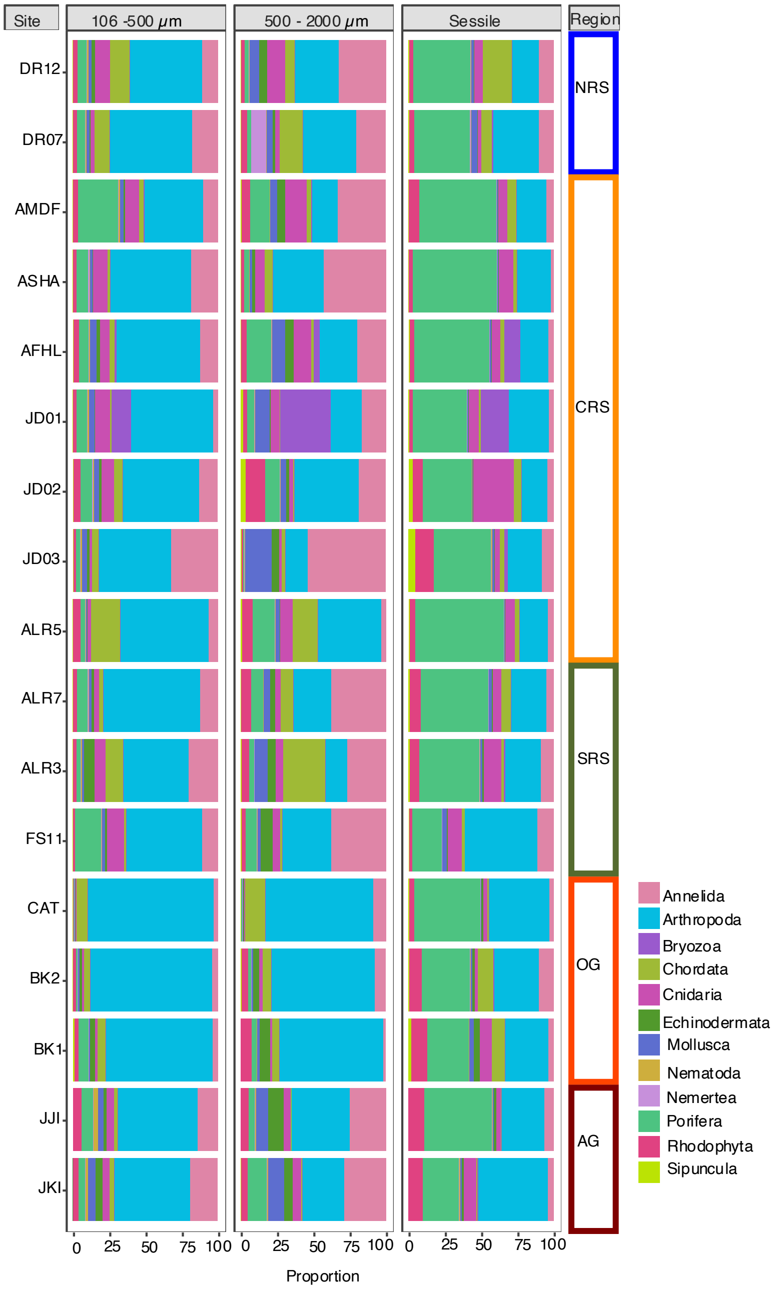

[Figure S-1] Cryptobenthic assemblage (mobile 106-500 µm, 500-2000 µm; sessile) composition plots with relative read abundance by phylum for each reef sampled using ARMS. Regions: NRS - northern Red Sea, SRS - southern Red Sea, CRS - central Red Sea, OG - Gulf of Oman, and AG – Arabian (Persian) Gulf.

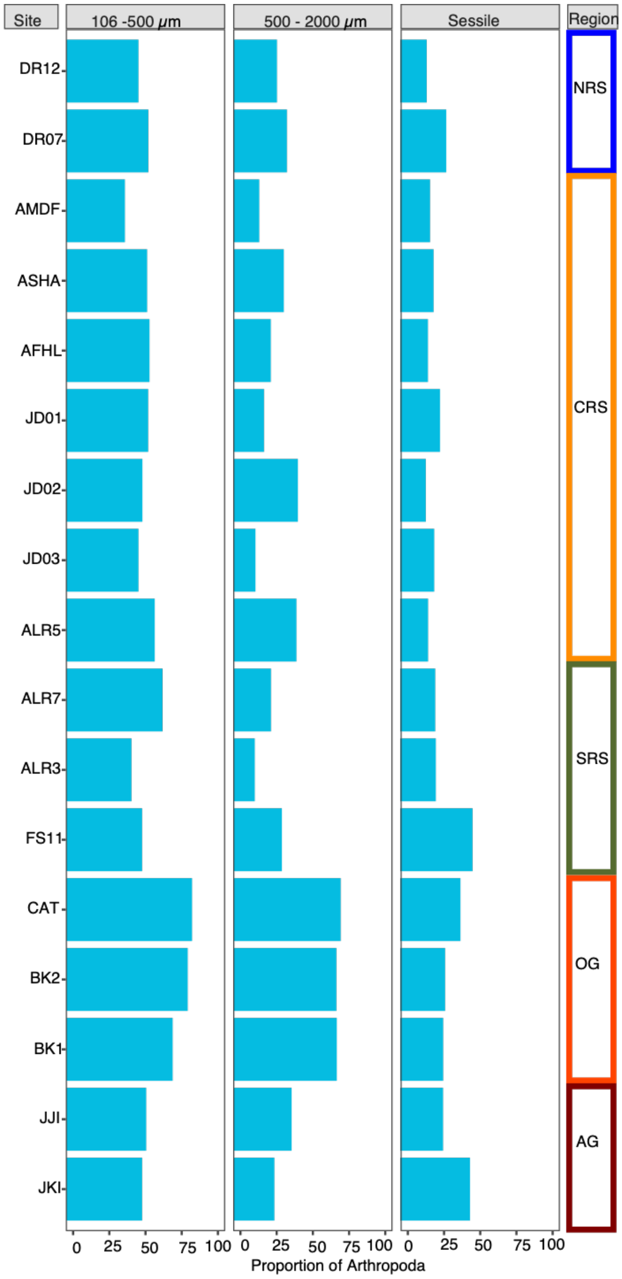

[Figure S-2] Relative read abundance of Arthropoda for each reef sampled using ARMS. Regions: NRS - northern Red Sea, SRS - southern Red Sea, CRS - central Red Sea, OG - Gulf of Oman, and AG – Arabian (Persian) Gulf.

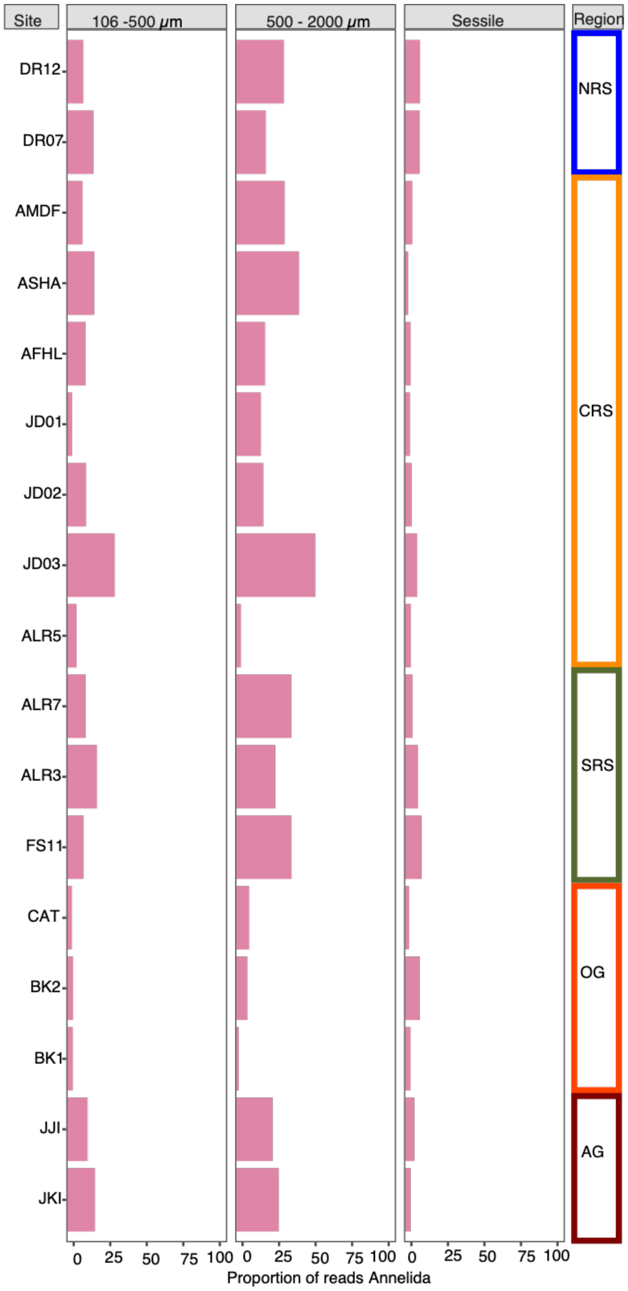

[Figure S-3] Relative read abundance of Annelida for each reef sampled using ARMS. Regions: NRS - northern Red Sea, SRS - southern Red Sea, CRS - central Red Sea, OG - Gulf of Oman, and AG – Arabian (Persian) Gulf.

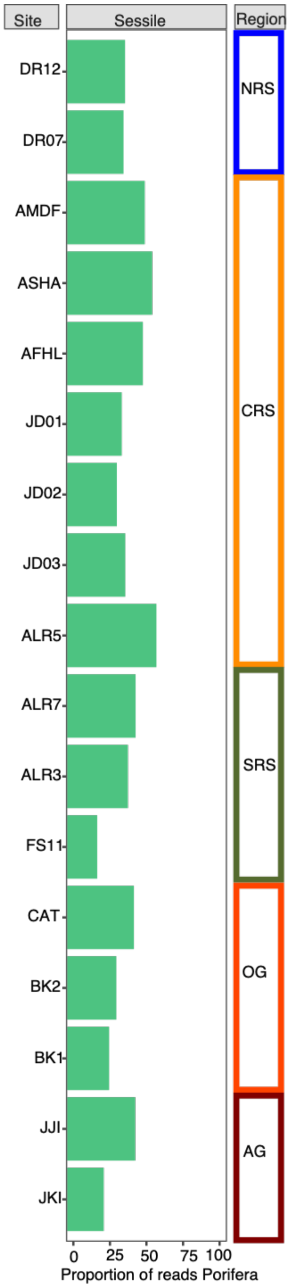

[Figure S-4] Relative read abundance of Porifera for each reef sampled using ARMS. Regions: NRS - northern Red Sea, SRS - southern Red Sea, CRS - central Red Sea, OG - Gulf of Oman, and AG – Arabian (Persian) Gulf.

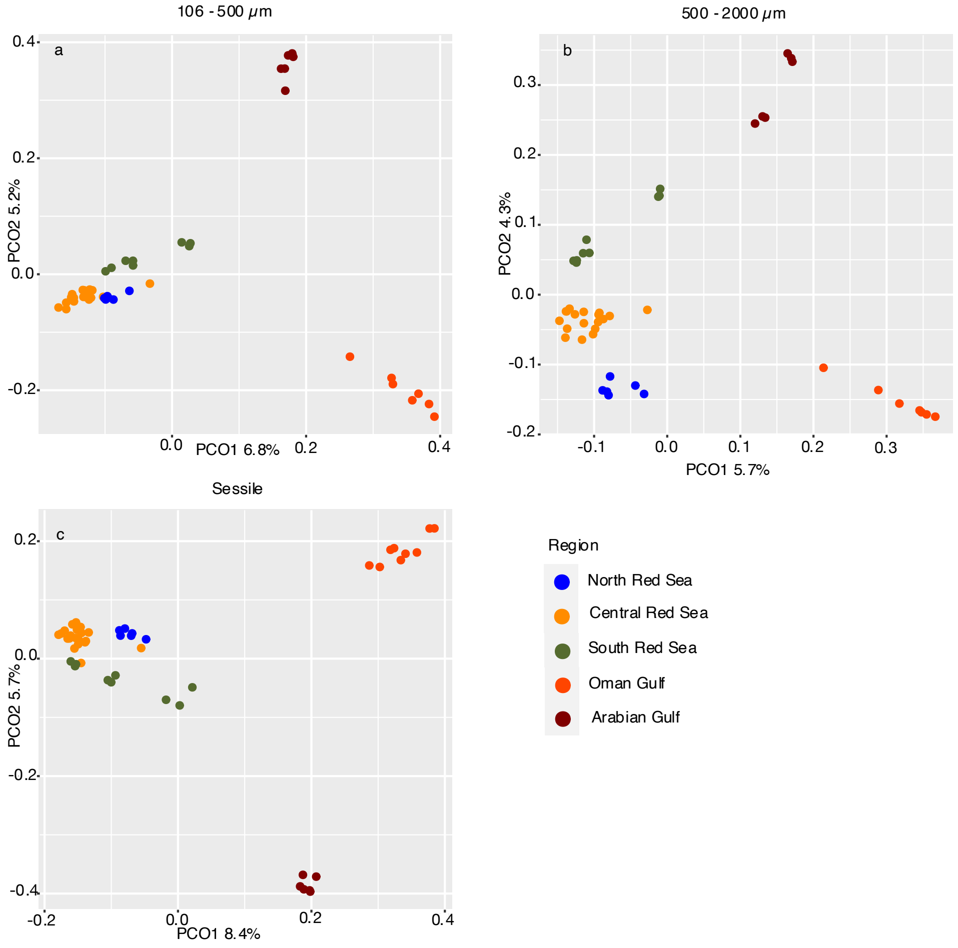

[Figure S-5] Principal coordinate analysis of the A) 106-500 µm, B) 500-2000 µm, and C) sessile fractions of the Jaccard dissimilarity matrix visualizing the differences in community composition between ARMS and regions.

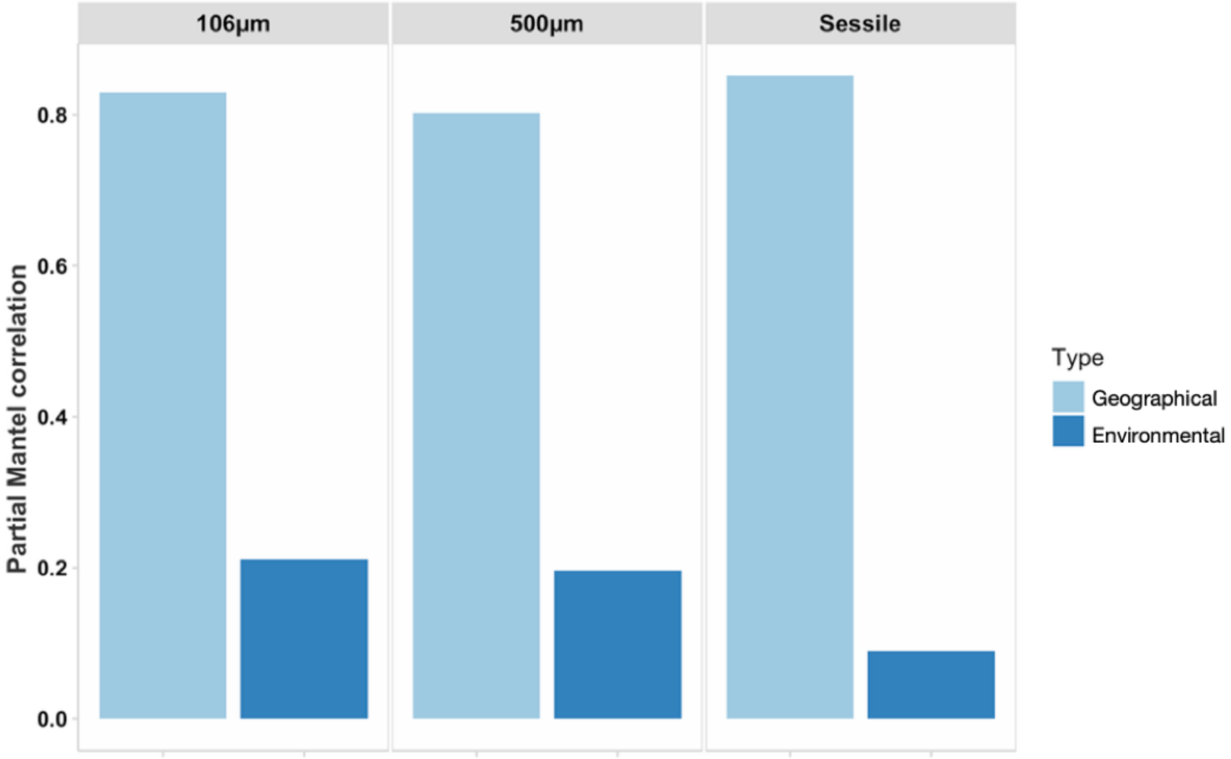

[Figure S-6] Correlations between community similarity with geographical and environmental distances. Partial mantel correlations showing the relative contribution of geographical and environmental distances in the community structure. 106 = 106-500 mobile fraction, 500 = 500-2000 mobile fraction, Sessile = Sessile fraction.
